# Right‐wing authoritarianism and perceptions that minoritized groups pose a threat: The moderating roles of individual‐ and country‐level religiosity and marginalization

**DOI:** 10.1111/bjso.12830

**Published:** 2025-01-16

**Authors:** Fahima Farkhari, Julian Scharbert, Lara Kroencke, Christin Schwarzer, Jonas F. Koch, Maarten H. W. van Zalk, Bernd Schlipphak, Mitja D. Back

**Affiliations:** ^1^ University of Münster Münster Germany; ^2^ University of Bonn Bonn Germany; ^3^ University Osnabrück Osnabruck Germany; ^4^ Joint Institute for Individualisation in a Changing Environment (JICE) University of Münster and Bielefeld University Münster Germany

**Keywords:** anti‐immigrant, deprivation, experience sampling method, minorities, perceived threat, political attitudes, political ideology

## Abstract

Right‐wing authoritarianism (RWA) refers to an adherence to conventional values and authorities with the power to penalize groups that are perceived to challenge the cohesion of ingroup norms. Correspondingly, RWA has repeatedly been linked to negative perceptions of minoritized groups, such as refugees or religious minorities. To investigate whether and how sociocultural factors add to and moderate how RWA influences perceptions that minoritized groups pose a threat (i.e. threat perceptions), we examined (a) the value of RWA, religiosity and perceived societal marginalization in predicting these threat perceptions across countries, (b) potential moderating effects of individual‐ and country‐level religiosity and marginalization on the RWA‐threat link and (c) the robustness of cross‐sectional findings when daily threat perceptions were assessed longitudinally. We used cross‐sectional survey data from Germany *N* = 1896; Study (1) and Europe *N* = 3227; Study (2) and global cross‐sectional and longitudinal daily diary data *N* = 3154 individuals; *N* >52,447 assessments; *N* = 41 countries; Study (3). Our studies point to the significance of contextual conditions and the generalizability of cross‐sectional findings to day‐to‐day assessments of threat perceptions.

## INTRODUCTION

There is a long tradition of associating right‐wing authoritarianism (RWA) with negative perceptions of minoritized groups (Adorno et al., 1950; Fromm, 1941). The now widely recognized conceptualization of RWA (Altemeyer, 1981) includes a rigid adherence to conventional values and authorities with the power to penalize groups that are perceived to challenge ingroup cohesion. Given that minoritized groups (e.g. refugees or religious minorities) are often perceived by the majority group as threatening to conventional values and norms, economic resources or safety, the robust link between RWA and such threat perceptions is no surprise. While studies on the link between RWA and negative perceptions of minoritized groups, including threat perceptions, abound (e.g. Cohrs & Stelzl, 2010; Dunwoody & McFarland, 2018; Onraet et al., 2021; Sibley & Duckitt, 2008), few studies have explored whether and how individual‐ and country‐level sociocultural factors add to and moderate the influence of RWA on threat perceptions. Against the backdrop of increasing refugee and right‐wing movements, we aimed to examine the relationship between RWA and perceptions that minoritized groups pose a threat while considering sociocultural factors. Specifically, we focused on threat perceptions with respect to ethnic, national or religious outgroups. According to the latest Global Trends report of the UNHCR, the number of refugees, asylum seekers, internally displaced and stateless people has been continually increasing over the past decade (UNHCR, 2024). While the global refugee crisis has triggered tremendous solidarity among some, it has provoked negative attitudes and perceptions of threat in others. The refugee crisis has been accompanied by increased anti‐immigration rhetoric in political and public discourse, particularly driven by right‐wing parties and promoting a normalization of racist discourse (e.g. Krzyżanowski, 2020). Although the effects of ethnic, religious and national (dis‐)similarity on outgroup perceptions seem to be smaller than public narratives suggested after the outbreak of war in Ukraine, there is evidence of the stable existence of diverging perceptions as a function of such a (dis‐)similarity (e.g. decreased willingness of accepting Muslim compared to Christian immigrants, refugees or asylum seekers in Europe; Bansak et al., 2023; De Coninck, 2020). In detail, the present work investigated (1) the roles of individual‐level RWA, religiosity and perceived societal marginalization (PSM) in predicting threat perceptions and (2) the potential moderating effects of individual‐ and country‐level religiosity and marginalization. We relied on three studies providing German, European and global data, comprising cross‐sectional data and longitudinal daily diary data. Not only do our studies provide a better understanding of the conditions that weaken or reinforce the RWA‐threat link, but they also test the generalizability of the RWA‐threat link when applied to more proximate day‐to‐day threat perceptions and across a wider range of country contexts.

### The prediction of threat perceptions from individual‐level RWA, religiosity and PSM

The relationship between RWA and threat perceptions is the subject of various theories, most prominently the dual‐process motivational model of ideology and prejudice (DPM; Duckitt, 2001; Duckitt & Sibley, 2017). This model links RWA with threat perceptions and prejudice towards social groups on the basis of two characteristics related to RWA: the ‘dangerous worldview’ and the intolerance of deviations (e.g. from norms and values). The RWA‐threat link is also considered in revised versions of the Intergroup Threat Theory (ITT; Stephan et al., 2016, pp. 260–261). As mentioned above, previous research found that RWA was reliably linked to threat perceptions and prejudice towards minoritized groups. In this study, using a large data set enabling high‐powered analyses and more precise estimates, we expected to extend the evidential basis and find that individual‐level RWA is a positive predictor of perceptions that minoritized groups pose a threat with moderate to very large effect sizes (Hypothesis 1).

While the RWA‐threat link is undisputed, the predictive value of sociocultural individual‐level factors both independent of and considering RWA is still unclear. Here, we focus on the examination of religiosity and PSM. While religiosity is still a central and widespread sociocultural factor in many countries (Joshanloo & Gebauer, 2020), its relationship with threat perceptions is yet unsolved. Existing theoretical assumptions about this relationship have been contradictory: While one central account suggests a positive link, arguing that religious individuals may feel that outgroups threaten their traditions, a contrasting account assumes a negative link, arguing that religious values (e.g. charity) require religious individuals to embrace all individuals as equals and support those in need of help. Previous empirical findings have been as inconsistent as theoretical assumptions (see, e.g. Benoit, 2021; Rowatt, 2019). Thus, we aimed to explore how religiosity is related to threat perceptions (Research Question 1; RQ1) using large cross‐sectional survey data gathered across multiple countries. Thereby, we examined the relationship between religiosity and threat perceptions both independent of and when controlling for RWA. The second sociocultural factor we focused on is PSM. PSM describes the degree to which individuals experience ‘people like them’ as being economically, culturally and politically marginalized (Bollwerk et al., 2022). While various indicators of subjective deprivation (e.g. group relative deprivation) have been associated with perceptions that minoritized groups pose a threat (Meuleman et al., 2020), the relationship between PSM and threat perceptions has yet to be explored. The construct of PSM and its corresponding measure were introduced only recently and have been studied only in the German context (Bollwerk et al., 2022; Bollwerk et al., 2024). Several lines of argumentation support the assumption that PSM should be positively related to perceptions that minoritized groups pose a threat. First, according to social identity theory, individuals strive for a positive self‐concept. Individuals high in PSM may engage in downwards intergroup comparisons in which outgroups are evaluated negatively to improve the perceived status of their own social group (see Hornsey, 2008). Second, cognitive perspectives on prejudice (e.g. Duckitt, 1992) would suggest that individuals require resources to cope with otherness, uncertainty and ambiguity, but marginalized individuals may lack these resources due to the various kinds of daily perceived threats and stress related to their perceived deprivation of resources. Moreover, their threat system should be generally activated more easily. All these considerations led us to expect a positive association between PSM and perceptions that minoritized groups pose a threat (Hypothesis 2). To move beyond Bollwerk et al.'s (2022, 2024) studies, we explored the relationship by investigating the link between PSM and threat perceptions across a range of countries, again, with large samples and both independent of and when controlling for RWA.

### RWA‐threat link: The moderating roles of individual‐level religiosity and PSM

To the best of our knowledge, previous theories and empirical studies have not examined individual‐level moderators of the RWA‐threat link. However, there are good reasons to assume that religiosity and PSM can explain variations in threat perceptions, not only in addition to RWA, but they might also interact with RWA. Traditionally, RWA is characterized by a desire to maintain order, traditions and the status quo, which facilitates threat perceptions regarding those perceived as different and disruptive to that order (Osborne et al., 2023). Some authors have argued for a singularity of religion as social identity and for its unique power in shaping psychological processes due to religious identity being grounded in faith and firm religious belief systems (Ysseldyk et al., 2010). On the basis of this argumentation, it is possible for the aforementioned desires and values to gain a particular (e.g. transcendent) significance for right‐wing authoritarian individuals if paired with religiosity. That is, religiosity may strengthen the positive link between RWA and threat perceptions due to religiously charged right‐wing authoritarian values. However, it is equally conceivable that due to religiosity exerting an identity‐promoting and psychologically stabilizing influence (see also Ysseldyk et al., 2010), religiosity may serve as a buffer against threat perceptions. Regarding PSM, opposing assumptions can also be made. PSM is accompanied by the perception that one has a societally low status, which might go along with a strong need to create positive distinctiveness. For this reason, higher levels of PSM might drive right‐wing authoritarian individuals to value, support and protect authoritarian values even more firmly. Furthermore, the constant daily experience of threat in individuals high in PSM may trigger the right‐wing authoritarian threat focus, leading to increased threat perceptions (see also Stecker et al., in press). Thus, individual‐level PSM could strengthen the RWA‐threat link. Alternatively, although less likely, it is also conceivable for the opposite pattern to appear, for example, if individuals increasingly engage in strategies to psychologically cope with their status quo with increasing levels of PSM (e.g. system‐justifying strategies; Jost & Hunyady, 2003).

We are not aware of studies that have examined whether individual‐level religiosity or individual‐level PSM explain variations in the RWA‐threat link and, if so, whether these variables have strengthening or weakening effects. Therefore, we explored the potential moderating role of individual‐level religiosity (RQ2) and individual‐level PSM (RQ3) in predicting threat perceptions from RWA across three studies and a range of countries.

### RWA‐threat link: Roles of country‐level religiosity and marginalization

We additionally aimed to understand how religiosity and marginalization at the country level influence the RWA‐threat link. Various arguments have previously been made for the relevance of cultural or societal factors in explaining threat perceptions. Earlier revisions of the ITT have discussed several cultural factors that could influence threat perceptions more generally, including individualism–collectivism, power distance and uncertainty avoidance (Stephan et al., 2009; pp. 47–48). However, later revisions removed this group of antecedents from the theory due to insufficient research on this topic (Stephan et al., 2016; p. 259; p. 273). Adorno et al. (1950) already commented more than 70 years ago that ‘personality can never be isolated from the social totality within which it occurs’ (p. 5), including the ‘individual's position in society as defined in economic and sociological terms’ (p. 8). In the DPM, Duckitt (2001) suggested that cultures and societies with high expressions in a dangerous worldview should be characterized by greater vulnerability to negative outgroup perceptions and prejudice. Despite arguments for variations in threat perceptions as a function of societal circumstances, it is yet unknown whether, how and which cultural factors influence the RWA‐threat link. The present work aimed to investigate the moderating roles of country‐level religiosity and marginalization in variations of the RWA‐threat link.

As in the analyses on the individual level, arguments for potential moderating influences of country‐level religiosity and marginalization can be made for both weakening or strengthening effects of RWA on threat perceptions. Typically, countries high in religiosity tend to support the preservation of traditions and the status quo (Norris & Inglehart, 2011). As a result, individuals high in RWA might feel more secure in more religious countries both psychologically and in their identities. Such perceived security and stability might serve as a buffer against threat perceptions, resulting in weaker RWA‐threat links in countries with high compared with low levels of religiosity. However, by contrast, it is also conceivable that the RWA‐threat link is stronger in religious countries, as right‐wing authoritarian individuals have more to lose in religious countries than in more secular ones. Similar assumptions can be made for country‐level marginalization. The European Migration Network (n.d.) defines marginalization as ‘a situation whereby a person is prevented (or excluded) from contributing to and benefiting from economic and social progress’. Accordingly, marginalization can encompass exclusion from diverse types of societal goods, such as income (equality), civil liberties and political participation. Marginalization typically affects minority groups (e.g. immigrants) to a greater extent than historically privileged groups (see, e.g. Eugster, 2018). As such, it supports and represents the maintenance of traditional structures and social hierarchies. Here, too, the possibility arises that a perception of security resulting from such a maintenance of the status quo will serve as a buffer against threat perceptions or that it will lead, alternatively, to the opposite effect due to fear of losing one's (historically privileged) status. In addition, in countries with high levels of marginalization, majority group members may be frequently reminded of existing social exclusion and the possibility of social relegation. Even if majority group members high in RWA are not directly affected by the marginalization, the frequent reminders of its existence and the possibility of being affected by it may nevertheless trigger their focus on the threats posed by minoritized groups. Overall, the questions of potentially moderating roles of country‐level religiosity (RQ4) and marginalization (RQ5) in the RWA‐threat link remain theoretically and empirically unanswered. This research is the first to address these questions using data from a global study that included >50 countries (of which 10 countries had *N* > 50 respondents).

### Correspondence of findings when using daily threat perceptions

Typically, threat perceptions and related constructs are measured cross‐sectionally. We are only aware of a few studies that have used a daily diary approach in the field of intergroup relations (e.g. Prati et al., 2022), at most including threat perceptions as a cross‐sectionally measured predictor (Van Acker et al., 2014) and moreover focusing on one specific country. Thus, the understanding of daily threat perceptions in everyday life and their predictors has yet to be explored. Moving beyond previous studies, in Study 3, we used global cross‐sectional and daily measurements of threat perceptions to attempt to replicate previous findings that were based on cross‐sectional measurements.

## METHODS

As we are committed to the standards of openness and transparency in science, we provide our study materials including anonymized data and analysis codes on the OSF (https://osf.io/2excn/). The research questions, hypotheses and analysis plan for Study 3 were preregistered.^1^ All deviations from the preregistration are documented.

## STUDY 1

Studies 1a and 1b employed identical measures and were both conducted in Germany.

### Sample and procedure

The Study 1a data were collected in summer 2019 via the online panel provider PsyWeb (original sample: *N =* 1003). The Study 1b data were collected in autumn 2019 via the online panel provider respondi AG (original sample: *N* = 1023). After data cleaning and the exclusion of respondents with a non‐Christian religious affiliation or non‐German citizenship (see Appendix A), *N*_Study1a_ = 923 and *N*_Study1b_ = 973 respondents remained in the sample.^2^

### Measures

To measure threat perceptions with respect to refugees, we used Landmann et al.'s (2019) 9‐item scale. Symbolic threat (e.g. ‘The refugees values and beliefs are not compatible with those of Germans’), realistic threat (e.g. ‘The refugees living here threaten Germany's economic situation’) and safety threat (e.g. ‘The refugees living here threaten public safety in Germany’; 1 = *strongly disagree* to 5 = *strongly agree*; *α*_Study1a_ = .95; *α*_Study1b_ = .96) were measured with three items each. To measure RWA, we administered Beierlein et al.'s (2014) German 9‐item Authoritarianism Short Scale (KSA‐3; e.g. ‘It is always best to do things the usual way’; 1 = *strongly disagree* to 5 = *strongly agree*; *α*_Study1a_ = .84; *α*_Study1b_ = .86), with the three RWA subdimensions each measured with three items. Religiosity was measured with the item ‘How religious do you consider yourself to be?’ (1 = *not at all religious* to 7 = *very religious*). To measure PSM, we used Bollwerk et al.'s (2022) 15‐item PSM scale.^3^ Economic PSM (e.g. ‘No matter how hard we work, people like me are not appreciated enough’), cultural PSM (e.g. ‘The customs, traditions and manners of people like me are less and less appreciated’) and political PSM (e.g. ‘Most politicians don't care what people like me think’; 1 = *strongly disagree* to 6 = *strongly agree*; *α*_Study1a_ = .94; *α*_Study1b_ = .94) were measured with five items each.

## STUDY 2

Whereas Study 1 was limited to Germany, Study 2 was conducted in four European countries. The examination of these different countries allowed for a test of the robustness and potential differences in the RWA‐threat link across societal contexts.^4^ Moreover, unlike in Study 1, we measured not only threat perceptions with respect to refugees but also with respect to Muslims. At the time of data collection, before the outbreak of war in Ukraine, most migrants and refugees in Europe were Muslims (Pew Research Center, 2017). Although there was also a substantial share of Christian migrants and (to a lesser degree) Christian refugees, the public debate focused almost exclusively on Muslim refugees. To examine whether we find differences when examining perceptions that refugees pose a threat compared to perceptions that Muslims pose a threat, we assessed both types of perceived threat in Study 2. However, as the correlation between these types of threats was very strong, we aggregated the measures into one measure of perceived threat.

### Sample and procedure

The data were collected in autumn 2020 under the responsibility of Kantar Deutschland (Berlin) via computer‐assisted telephone interviewing (CATI). The original sample comprised *N* = 5011 (*N*_FRA_ = 1208; *N*_GER_ = 1402; *N*_POL_ = 1200; *N*_SWE_ = 1201) respondents. After data cleaning and the exclusion of respondents with a non‐Christian religious affiliation or a migratory background (see Appendix A), *N* = 3227 respondents remained in the sample (*N*_FRA_ = 723; *N*_GER_ = 940; *N*_POL_ = 865; *N*_SWE_ = 699).

### Measures

In Study 2, we measured threat perceptions with respect to Muslims with six items, with two items each assessing symbolic, realistic and safety threats (self‐generated items based on the refugee threat scale by Landmann et al., 2019, see Appendix A; 1 = *do not agree at all* to 6 = *fully agree*; *α* = .90). Threat perceptions with respect to refugees were assessed with three items, each measuring one of the three threat dimensions (adapted from Landmann et al., 2019; e.g. ‘The refugees' values and beliefs are incompatible with those of Germans’; see Appendix A; 1 = *do not agree at all* to 6 = *fully agree*; *α* = 84). We aggregated Muslim and refugee threat into one measure (*α* = .92). To measure RWA, we administered three items from the Authoritarianism Short Scale (KSA‐3; Beierlein et al., 2014), with one item each measuring the three RWA subdimensions (1 = *do not agree at all* to 6 = *fully agree*; *α* = .55). Religiosity was measured with the item ‘How religious do you consider yourself to be?’ (1 = *not religious at all* to 6 = *deeply religious*). To measure PSM, we used six items from the PSM scale (Bollwerk et al., 2022), with two items each measuring the economic, cultural and political subdimensions (1 = *do not agree at all* to 6 = *fully agree*; *α* = .87).

## STUDY 3

Study 3 moved beyond the previous studies by including a global data set and not only cross‐sectional but also daily measurements of threat perceptions.

### Sample and procedure

The data were taken from the Coping with Corona (CoCo) project (Scharbert et al., 2023; OSF: https://osf.io/dhmpy/) and collected via the software formr (Arslan et al., 2020) in 71 countries. The data were collected between October 2021 and August 2022 within a time frame of a few weeks for each participant. The participants first filled out an online presurvey in which they provided demographic information and responded to a range of trait items including those central to our study. The day after the presurvey was complete, an experience sampling phase began. In this phase, participants were invited to respond to brief state surveys multiple times per day and daily surveys once per day after 7 p.m. over a period of 4 weeks. Finally, a post‐survey assessed largely the same information as the presurvey. The present study used only data from the cross‐sectional presurvey and the daily diary surveys. The original sample comprised *N* = 7490 participants from 71 countries with *N* > 73,295 daily assessments of threat perceptions. Data cleaning was performed as recommended by the authors who coordinated the data collection (Scharbert et al., 2023; see Appendix A). After data cleaning and the exclusion of participants who indicated having a migratory background or were not citizens of the country in which they resided, *N* = 3154 participants from 41 countries with *N* = 52,447 total daily measurements remained in the sample (see Table A1 in Appendix A for details).^5^

### Measures

#### Individual‐level measures

Trait‐level threat perceptions were measured with six items introduced by the instructions, ‘The following statements refer to people in your country who belong to a different ethnic, religious, or national group than yourself. For each statement, please indicate the extent to which you agree with that statement’. Symbolic, realistic and safety threats from outgroups were measured with two items each (self‐generated items based on the refugee threat scale by Landmann et al., 2019, see Appendix A; 1 = *strongly disagree* to 6 = *strongly agree*; *α* = .95). Daily threat perceptions were measured with the item, ‘How threatened have you felt by people who belong to a different ethnic, religious, or national group than yourself?’ in each daily survey (1 = *not at all threatened* to 10 = *extremely threatened*). Participants were asked to rate the question with respect to the past day. RWA was measured with a 9‐item short RWA scale (KSA‐3; Beierlein et al., 2014; 1 = *do not agree at all* to 5 = *completely agree*; *α* = .85). Religiosity was measured with the item ‘How religious do you consider yourself to be?’ (1 = *not at all religious* to 10 = *very religious*). PSM was measured with six items that measured economic, cultural and political PSM with two items each (Bollwerk et al., 2022; 1 = *strongly disagree* to 6 = *strongly agree*; *α* = .89).

#### Country‐level measures

We used Joshanloo and Gebauer's (2020) country‐level religiosity index as our measure of country‐level religiosity. This index reflects the percentage of people per country who affirmed the Gallup World Poll item ‘Is religion an important part of your daily life?’ We considered countries with <50% as low in religiosity, countries with 50%–75% as moderate in religiosity, and countries with >75% as high in religiosity. The choice of these thresholds was based on two objectives: first, to categorize the countries into three levels of religiosity without artificially assigning countries with very similar levels of religiosity to different levels of religiosity; second, to maximize the distance between the country groups without making the groups too small. As a result, 21 countries were considered low (*N* = 1500), six countries moderate (*N* = 1041) and 14 countries high in religiosity (*N* = 613; see Table A1 in Appendix A for individual indices for each country).

To assess country‐level marginalization, we relied on various country‐level indices to approximate economic, cultural and political marginalization on a country level. Here, we briefly describe the indices. A more detailed description can be found in Appendix A. We used the Gini index (income distribution) as an indicator of country‐level economic marginalization (World Bank, 2022). This index measures the degree to which the distribution of income among individuals or households is distributed equally within a country. To assess country‐level cultural marginalization, we used ratings of civil liberties as reported in the ‘Freedom in the World’ (Freedom House, 2022a, 2022b, 2022c). The assessments were conducted across four subcategories, namely, freedom of expression and beliefs; associational and organizational rights; rule of law; personal autonomy and individual rights. To measure country‐level political marginalization, we relied on the assessment of political participation as was conducted for the generation of the ‘Democracy Index’ (Economist Intelligence Unit, 2022). The political participation category covers nine indicators, including voter participation, integration of minoritized groups into the political process, political interest and membership in political parties and organizations. If necessary, we recoded and transformed the scores on an index so that the scales ranged from 0 to 100 for all three indices with higher values corresponding to higher marginalization. To obtain a scale for overall country‐level marginalization, we aggregated the scores on the chosen indices of country‐level economic, cultural and political marginalization into a single mean score (*α* = .70). Subsequently, we categorized the countries as low (<25), moderate (25–40) or high (>40) in marginalization. The choice of these thresholds was based on the same criteria as for country‐level religiosity. As a result, 19 countries were considered low (*N* = 2029), 14 countries moderate (*N* = 754) and 8 countries high in marginalization (*N* = 371; see Table A1 Appendix A for individual indices for each country).^6^

## RESULTS

Table B1 in Appendix B present the descriptive statistics, including sample characteristics. In the following, we report the analytic procedure and results of the analyses separately for each set of research questions and hypotheses. All analyses were conducted in R (R Core Team, 2021).

### How are individual‐level RWA, religiosity and PSM associated with threat perceptions?

Figure 1, 2, 1‐3 presents how individual‐level RWA, religiosity and PSM were correlated with threat perceptions with respect to the minoritized groups in Studies 1–3.^7^ For Study 3, we present the correlations for both trait threat perceptions (in black) and daily threat perceptions (in green). To calculate the latter, we used the respondents' average daily threat perceptions. Using the R package ‘metafor’ (Viechtbauer, 2010), we also derived meta‐analytic correlations, calculating sample size‐weighted correlations across the individual samples in Studies 1–3 (in Study 3, only for countries with *N* > 50, see Figure 1, 2, 1‐3; meta‐analytic effect sizes were coloured blue for trait threat and green for daily threat). In all three studies, we found significant positive correlations between RWA and threat perceptions. Using Funder and Ozer's (2019) and Gignac and Szodorai's (2016) effect size guidelines, we found meta‐analytic effects ranging from large to very large for trait threat (*r*_Study1_ = .57 [.52, .63]; *r*_Study2_ = .39 [.35, .43]; *r*_Study3_ = .49 [.44, .54]) and a medium‐sized meta‐analytic effect for daily threat (*r*_Study3, daily_ = .26 [.18, .34]). We found no consistent correlational pattern between religiosity and threat perceptions. In line with most of the individual samples, two meta‐analyses showed small‐ to medium‐sized positive correlations (*r*_Study1_ = −.05 [−.08, −.02]; *r*_Study2_ = .19 [.17, .22]; *r*_Study3_ = .22 [.20, .23]; *r*_Study3, daily_ = .10 [.08, .12]). In nearly all the individual samples, PSM was positively related to threat perceptions with large to very large meta‐analytic effect sizes (*r*_Study1_ = .46 [.42, .50]; *r*_Study2_ = .44 [.40, .49]; *r*_Study3_ = .30 [.27, .33]; *r*_Study3, daily_ = .20 [.14, .26]).

**FIGURE 1:**
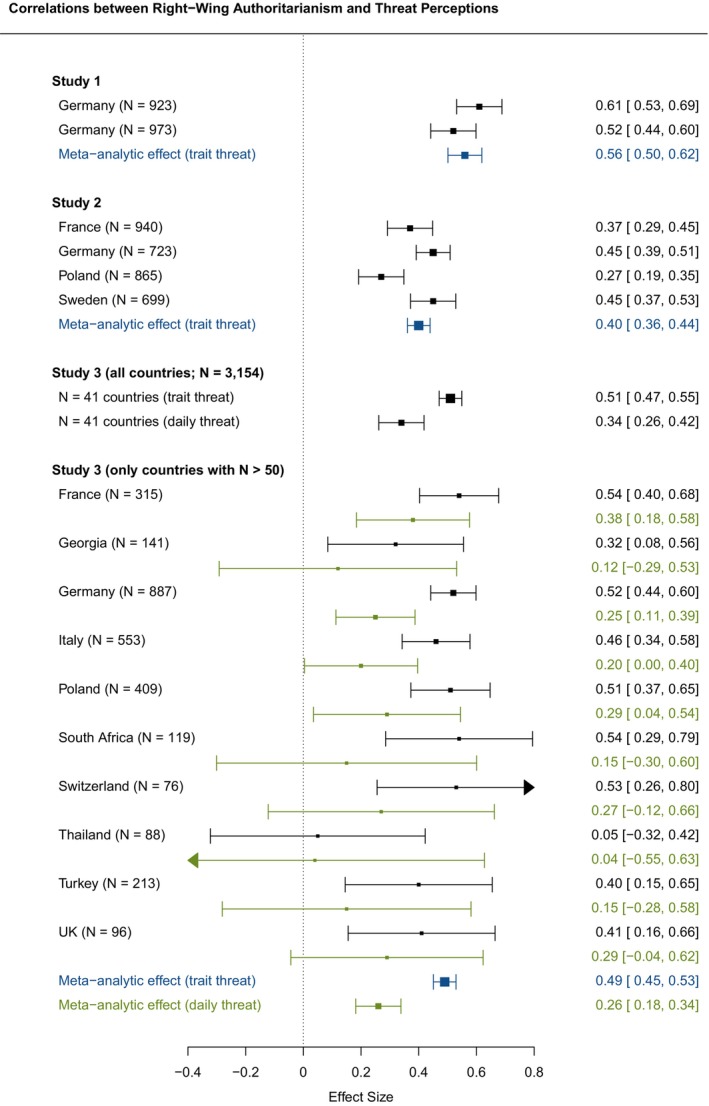


**FIGURE 2:**
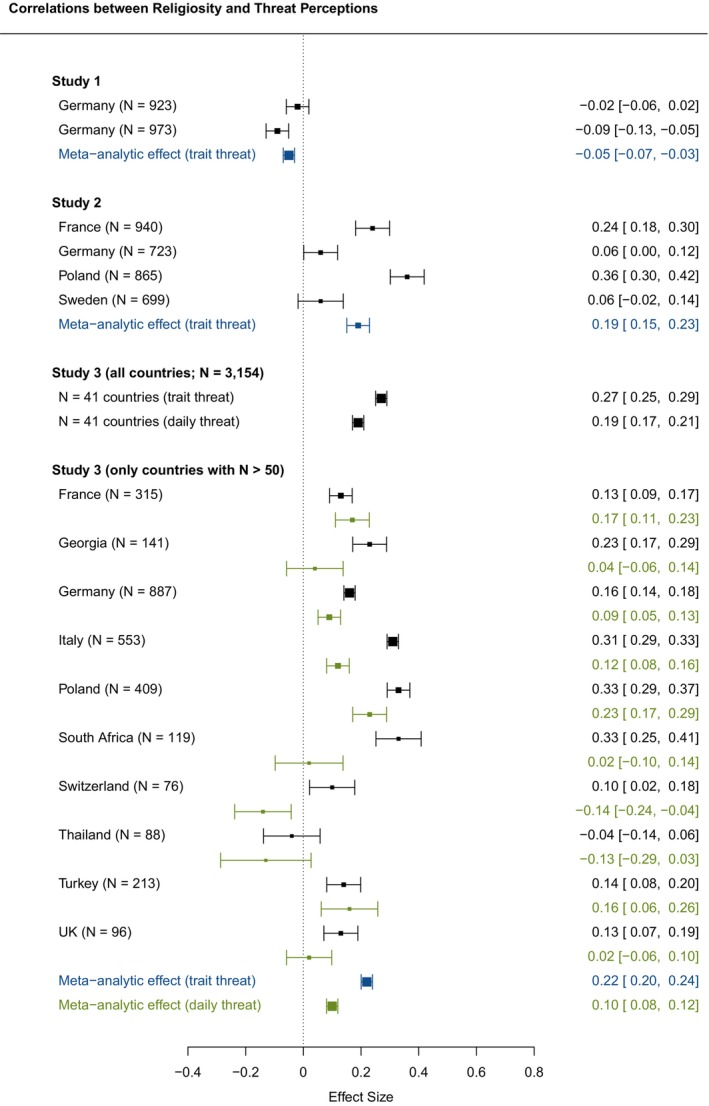


**FIGURE 1‐3:**
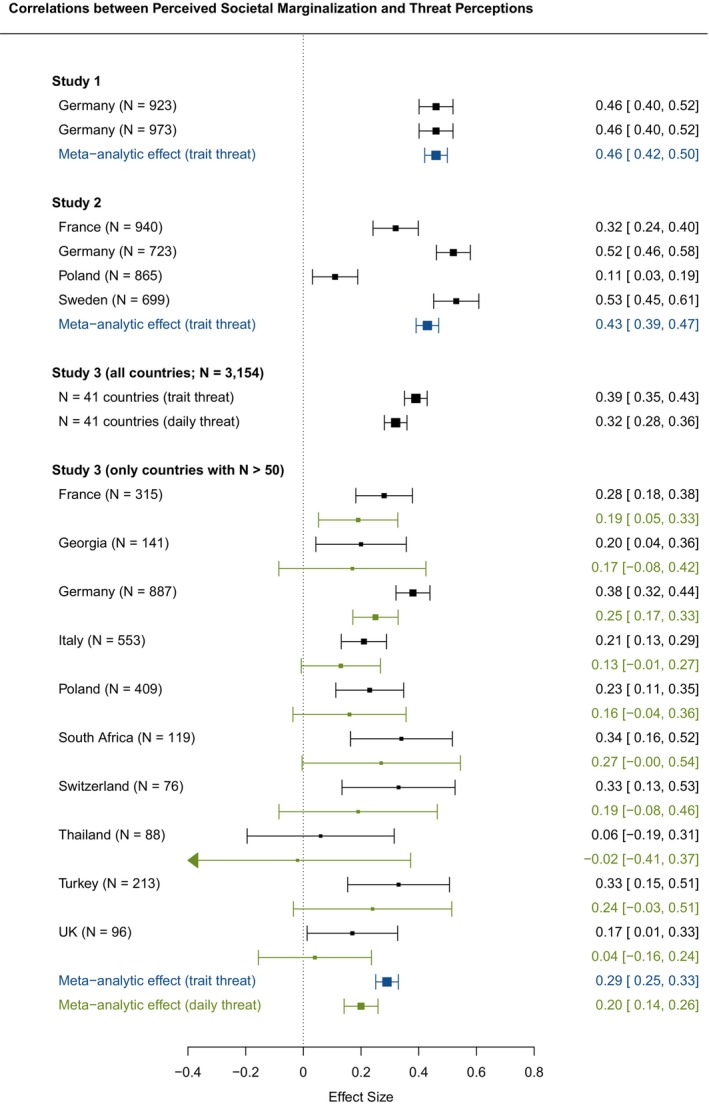
Bivariate correlations of RWA, religiosity and PSM with threat perceptions (Studies 1–3). UK, United Kingdom. Arrows at the end of a line indicate that the respective boundary of the 95% confidence interval exceeds the area of the plot. Coefficients from the individual studies involving measurements of trait threat are coloured black. Sample size‐weighted meta‐analytic correlations across the individual samples from the respective study involving trait threat are coloured blue. The coefficients from the individual studies involving daily threat (only in Study 3 and for countries with *N* > 50 participants) and the sample size‐weighted meta‐analytic correlations across these studies are coloured green.

To understand the unique value of each predictor in explaining variation in threat perceptions, we calculated regression analyses, first, including all three predictors jointly in the regression model, and second, adding sociodemographic control variables. For the analyses using cross‐sectional measurements of threat perceptions (Studies 1–3), we estimated multiple linear regression models (MLR). Continuous predictors and the outcome variable were *z*‐standardized prior to the analyses. For the analyses using daily measurements of threat perceptions (Study 3), we estimated multilevel models (MLMs). The MLMs were estimated with daily‐level observations of threat perceptions on Level 1 nested within individuals on Level 2. Individual‐level RWA, religiosity and PSM were measured cross‐sectionally; therefore, these observations were made at the individual level (level 2). As all predictors were measured on level 2, we predicted individual differences in the intercept (i.e., person mean) of daily threat perceptions. Continuous predictors were mean‐centred prior to the analyses. The multilevel analyses were computed with the lme4 package (Bates et al., 2015) and the default ML estimator REML (restricted maximum likelihood estimation).

Table B2 presents the results of analyses (MLR, MLM) in which all three predictors were included in the models and Table B3 those additionally including sociodemographic control variables (both in Appendix B). Underlining the unique values of RWA, PSM and religiosity in predicting threat perceptions, most effects remained significant with a similar order in the predictive values.^8^ In sum, whereas, overall, we did find variation in the effect sizes, RWA and PSM emerged as robust predictors of threat perceptions with respect to minoritized groups across countries. Religiosity showed an inconsistent pattern but was positively related to threat perceptions in most of the countries. Daily threat perceptions appeared to be similarly related to RWA, religiosity, and PSM as trait threat perceptions.

### Do individual‐level religiosity and individual‐level PSM moderate the association between threat perceptions and individual‐level RWA?

Table 1 presents RWA's interactions with individual‐level religiosity and individual‐level PSM in predicting trait threat based on moderated MLR (Studies 1–3) and daily threat based on MLMs (Study 3). The results of the analyses including sociodemographic control variables resulted in essentially the same coefficients. Across the six individual samples in Studies 1 and 2, we found that the RWA‐threat link was moderated by individual‐level religiosity in only one sample, namely, in the Polish sample in Study 2. The interaction was positive and weak (*β* = .09 [.03, .15], *p* = .002). Simple slope analyses (see Table B4 in Appendix B) revealed that, in that sample, individuals one standard deviation (SD) below the mean in individual religiosity showed a moderately strong relationship between RWA and threat perceptions (*β* = .23 [.15, .31], *p* < .001), whereas individuals one SD above the mean showed a very strong relationship (*β* = .40 [.32, .48], *p* < .001). In the overall sample in Study 3, the interaction was significant in the trait threat analyses (*β*_Study3, trait_ = .04 [.00, .08], *p* = .029; simple slopes: *β* = .56 [.50, .62] compared with *β* = .65 [.59, .70], both *p* < .001), but non‐significant in the daily threat analyses (*b*_Study3, daily_ = 0.02 [−0.01, 0.04], *p* = .264, see Table 1).

**TABLE 1:** Moderating effects of individual‐level religiosity or perceived societal marginalization on the RWA‐threat link (Studies 1–3).

	Study 1		Study 2				Study 3	
	Germany	Germany	France	Germany	Poland	Sweden	*N* = 41 countries	*N* = 41 countries
	*β*	*β*	*β*	*β*	*β*	*β*	*β*	*b* (MLM)
RWA	.**61**	.**52**	.**40**	.**45**	.**20**	.**53**	.**61**	**0.75**
	[.56, .67]	[.47, .58]	[.32, .48]	[.39, .51]	[.13, .27]	[.45, .61]	[.57, .65]	[0.67, 0.84]
Religiosity	−.04	**−.11**	.**21**	.01	.**29**	.08	.**14**	**0.05**
	[−.09, .01]	[−.17, −.06]	[.12, .30]	[−.04, .07]	[.23, .35]	[−.01, .18]	[.10, .18]	[0.03, 0.07]
RWA*Religiosity	−.02	−.03	.00	−.02	.**09**	.02	.**04**	0.02
	[−.07, .03]	[−.08, .02]	[−.08, .08]	[−.08, .03]	[.03, .15]	[−.07, .11]	[.00, .08]	[−0.01, 0.04]
RWA	.**49**	.**40**	.**36**	.**33**	.**28**	.**39**	.**40**	**0.65**
	[.44, .54]	[.34, .45]	[.28, .44]	[.28, .38]	[.21, .36]	[.31, .47]	[.37, .43]	[0.57, 0.73]
PSM	.**25**	.**30**	.**24**	.**42**	.**08**	.**55**	.**24**	**0.35**
	[.20, .30]	[.25, .36]	[.15, .32]	[.36, .47]	[.01, .15]	[.47, .63]	[.21, .26]	[0.30, 0.40]
RWA*PSM	.**11**	−.03	.07	.02	−.02	.**08**	.**09**	**0.10**
	[.07, .16]	[−.08, .02]	[−.01, .15]	[−.03, .07]	[−.09, .04]	[.00, .16]	[.06, .11]	[0.04, 0.16]
*N*	923	973	723	940	865	699	3154	3154

*Note*: Bold numbers represent significant coefficients (*p* < .05). The coefficients represent coefficients from the moderated multiple linear regression analyses in Studies 1, 2 and 3 (trait threat); RWA, religiosity, PSM and threat perceptions were *z*‐standardized prior to the analyses. The coefficients represent coefficients from moderated multi‐level modelling for Study 3 (daily threat); RWA, religiosity and PSM were mean‐centred prior to the analyses. In Studies 1a and 1b, threat perceptions were measured with respect to refugees. In Study 2, threat perceptions were measured with respect to refugees and Muslims and aggregated into one measure. In Study 3, threat perceptions were measured with respect to people in one's own country who belong to a different ethnic, religious or national group than oneself.

Abbreviations: *N*, total sample size; PSM, perceived societal marginalization; RWA, right‐wing authoritarianism.

Regarding the moderating effect of individual‐level PSM, in the six samples from Studies 1 and 2, we found that two samples had significant, positive, and very small‐ to small‐sized effects: the German sample from Study 1a (*β* = .11 [.07, .16], *p* < .001) and the Swedish sample from Study 2 (*β* = .08 [.00, .16], *p* < .001). In these two samples, individuals with an individual‐level PSM one SD below the mean (*β* = .13 [.06, .20], *p* < .001 for the German sample; *β* = .44 [.34, .54], *p* < .001 for the Swedish sample) showed a weaker relationship between RWA and threat perceptions compared with those one *SD* above the mean (*β* = .36 [.29, .43], *p* < .001 for the German sample; *β* = .58 [.48, .68], *p* < .001 for the Swedish sample). In the overall sample in Study 3, the moderating effect of PSM was significant, positive and small in size for both trait threat (*β*_Study3, trait_ = .09 [.06, .14], *p* < .001; simple slopes: *β* = .30 [.26, .34] compared with *β* = .48 [.44, .52], both *p* < .001) and daily threat (b_Study3, daily_ = 0.10 [0.04, 0.16], *p* < .001; simple slopes: *b* = 0.50 [.0.38, 0.62] compared with *b* = 0.75 [0.65, 0.85], both *p* < .001).

Thus, overall, we found little evidence that individual‐level religiosity moderated the RWA‐threat link and some evidence that individual‐level PSM strengthened the RWA‐threat link. The effect sizes were small. Furthermore, we did not find consistent effects across samples from the same country in our three studies.

### Do country‐level religiosity and country‐level marginalization moderate the association between threat perceptions and individual‐level RWA?

**FIGURE 4:**
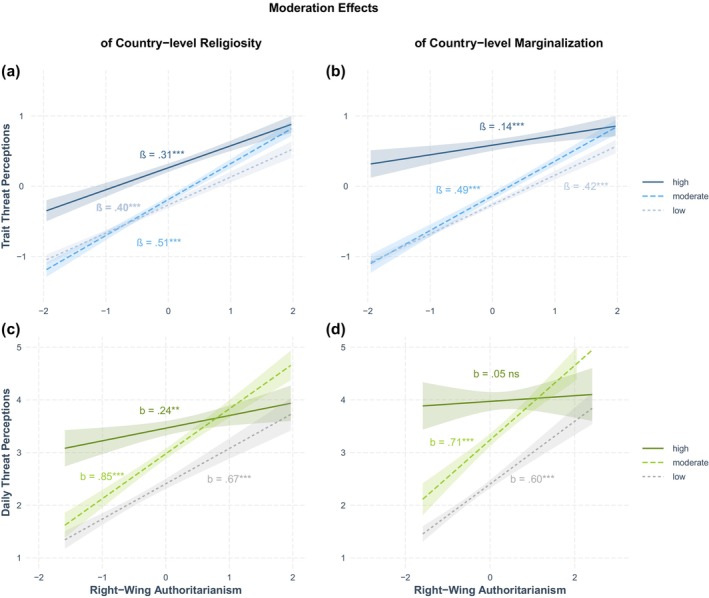
Moderating effects of country‐level religiosity or marginalization on the RWA‐threat link (Study 3). ns, not significant; SD, standard deviation. The coefficients represent the simple slopes of moderated multiple linear regression analyses when trait threat was predicted (*β*; a, b); right‐wing authoritarianism (RWA) and trait threat were *z*‐standardized prior to the analyses. The coefficients represent the simple slopes of multilevel modelling when daily threat was predicted (*b*; c, d); RWA was mean‐centred prior to the analyses. **p < .01 ***p < .001.

As Figure 1, 2, 1‐3 show, the associations between RWA and threat perceptions were relatively strong but also varied considerably across countries. Thus, we used the data from Study 3 to explore whether country‐level religiosity and marginalization moderated the association between RWA and threat perceptions. Figure 4 depicts RWA's interactions with country‐level religiosity (left panels) and country‐level marginalization (right panels) in predicting threat perceptions on the basis of trait threat (Panels a and b) and daily threat (Panels c and d). We found that both country‐level religiosity and country‐level marginalization moderated the RWA‐threat link (see Table B5 in Appendix B; results of models additionally including control variables were comparable). For both trait threat and daily threat, we found that the positive slope of the RWA‐threat link was most pronounced in countries with moderate religiosity (*β*_trait_ = .51 [.46, .56], *p <* .001; b_daily_ = 0.85 [0.72, 0.99], *p* < .001), followed by countries with low religiosity (*β*_trait_ = .38 [.35, .44], *p <* .001; b_daily_ = 0.67 [0.54, .079], *p <* .001). The weakest slope was found for countries with high religiosity (*β*_trait_ = .31 [.15, .38], *p <* .001; b_daily_ = 0.24 [0.06, 0.42], *p* = .008). All differences in slopes were significant for trait threat (*p* ≤ .028) and for daily threat (*p* < .05, see Table B5). Regarding country‐level marginalization, we also found that countries with moderate levels (*β*_trait_ = .49 [.44, .55], *p <* .001; b_daily_ = 0.71 [0.55, 0.86], *p <* .001) and low levels (*β*_trait_ = .42 [.38, .46], *p <* .001; b_daily_ = 0.60 [0.49, 0.70], *p <* .001) showed more pronounced slopes; in countries with high marginalization, the RWA‐threat link was substantially weaker for trait threat (*β*_trait_ = .14 [.06, .21], *p* = .001) and non‐significant for daily threat (*b*_daily_ = 0.05 [−0.17, 0.28], *p* = .633). All differences in slopes were significant for trait threat (*p* ≤ .026). Except for the difference in slopes for moderate compared with low country‐level religiosity (*b*_daily_ = 0.11, [−0.08, 0.30], *p* = .243), all differences in slopes were also significant for daily threat (*p* < .001; see Table B5). Thus, the RWA‐threat link appeared to be weaker in countries with high compared with moderate or low religiosity or marginalization. Results from preregistered supplementary analyses in which we applied alternative analytic approaches were largely consistent with these findings but more difficult to interpret due to low statistical power. They are summarized in Appendix C.

## DISCUSSION

The present research examined the RWA‐threat link across three studies that included cross‐sectional survey data and intensive longitudinal daily diary data across multiple countries. These data enabled us to extend the existing evidential basis on individual‐level RWA as a reliable predictor of majority groups' perceptions that minoritized groups pose a threat: We found large to very large meta‐analytic effect sizes across countries, even when additionally considering individual‐level religiosity and PSM. We additionally explored the unique predictive value of individual religiosity, for which previous theoretical and empirical work has been contradictory (see Benoit, 2021; Rowatt, 2019), as well as of PSM, a recently introduced construct (Bollwerk et al., 2022) that refers to perceived economic, cultural and political marginalization of one's own social group. We found that both religiosity and PSM were positive predictors of threat perceptions. Meta‐analytic effect sizes were moderate to very large for PSM and small to moderate for religiosity.

To add to the very thin amount of research on moderators of the RWA‐threat link, we additionally investigated both individual‐ and country‐level religiosity and PSM as potential moderators of the RWA‐threat link. Although our data did not find consistent support of a moderating effect of individual‐level religiosity or perceived societal marginalization, there was some evidence of a weak moderating influence of both variables. That is, in some samples, we found that individual‐level religiosity or PSM strengthened the positive RWA‐threat link. As these effects were weak and could not be replicated across studies in samples from the same country, this relationship requires further exploration. Whereas our data leave no doubt that individual‐level RWA is a strong overall predictor of majority groups' perceptions of threat from minoritized groups, the effect sizes still varied considerably across the individual samples.

The discussion on the relevance of cultural or societal factors in explaining threat perceptions is not new (Duckitt, 2001; Stephan et al., 2009), yet research had not yet identified whether, how, and which sociocultural factors influence the RWA‐threat link. Therefore, we additionally examined the moderating roles of religiosity and marginalization on the country level and found some evidence that the positive RWA‐threat link was weaker in countries with higher compared with moderate or lower levels of religiosity or marginalization. Stated differently, RWA is more predictive of majority groups' perceptions of threat from minoritized groups in less religious countries or countries with less marginalization. It therefore generally appears that contextual factors do play a role in shaping the RWA‐threat link. However, we still do not know why this may be the case. Earlier, we argued for potential moderating effects in both directions (strengthening and weakening). Arguments for a weakening effect of country‐level religiosity have focused on the possibility that right‐wing authoritarian individuals feel psychologically more secure in more religious countries, as more religious countries are more inclined to preserve traditions and the status quo than less religious countries (Norris & Inglehart, 2011). The perceived security and stability that might result from preserving traditions might equip right‐wing authoritarian individuals with psychological resources that protect them from perceiving minoritized groups as threatening. Arguments for a weakening effect of country‐level marginalization have focused on the possibility that, in countries with higher levels of marginalization, right‐wing authoritarian individuals perceive a maintenance of traditional structures and social hierarchies and consequently feel more secure as it should be the minoritized groups that are particularly excluded from societal goods in those countries (see Eugster, 2018). Again, the argument is that such perceptions of security and stability may serve as a buffer against threat perceptions. Our contrary assumptions that right‐wing authoritarian individuals from the majority group may perceive higher threat in countries high in religiosity or marginalization due to the fear of losing the stability that still exists (including their historically privileged status) was not supported by our data.

A central aim of our work was to investigate the extent to which the relationships under study could be replicated when threat perceptions were measured in everyday life in comparison with cross‐sectional measurements. The investigation of perceived threat, including its facets, has a decades‐long tradition in ITT (Stephan & Stephan, 2000) and beyond (Duckitt, 1992). However, to our knowledge, there is no research on daily perceived threat from outgroups, moreover, no research has examined these daily threat perceptions in relation to RWA. Using data from a global experience sampling study including cross‐sectional and daily diary surveys, we were able to find that the relationships between RWA and threat perceptions were weaker but generally similar when daily threat perceptions were used instead of cross‐sectional (trait) threat perceptions. That is, it seems that RWA is similarly predictive of the threat that individuals report feeling over the past day repeatedly across a period of several weeks compared with the threat that they indicate perceiving when asked more generally at one specific point in time. Some versions of the ITT distinguish between perceived threats to the group as a whole and perceived threats to individual members of a group. Our surveys covered the former in the cross‐sectional measurements of threat (referring to perceived threats to the country) and the latter in the daily measurements of threat (referring to perceived threats to oneself). Weaker effects in predicting daily threat perceptions based on RWA, religiosity or PSM, which we found in comparison with predictions of trait threat perceptions may result from these differences in operationalization. Potentially, our predictors are more explanatory of perceived threats to the collective (e.g. one's country) than of perceived threats to oneself. However, it is also possible that the predictors are more predictive of threats that are believed to exist (as measured with the cross‐sectional measure) compared with threats that are experienced on specific days (as measured with the daily measure). In addition, the daily measure did not differentiate between types of threat (i.e. symbolic, realistic, safety). It would be useful if future research measured all types of threat perceptions with both cross‐sectional and daily surveys. Comparable findings when using trait threat compared with daily threat were also found for the moderator analyses (examining individual‐ and country‐level religiosity and marginalization).

The Study 3 data were unique not only in that they provided daily data but also in that they covered a range of countries that differed in country‐level religiosity and marginalization, thereby offering us the unique opportunity to examine the potential roles these contextual factors may play in shaping the RWA‐threat link. However, our data were limited to one sample per country, had varying sample sizes across countries and despite covering comparably diverse samples were still dominated by WEIRD countries, thus impeding the understanding of contextual variability and generalizability of findings within or across countries and cultures (Rad et al., 2018). Future research should replicate the present country‐level investigation by covering more countries with larger and more diverse samples that are more equally distributed across countries.

Another noteworthy point is that our global study ran for a time frame of approximately 1 year, with individual participation taking place within a time span of a few weeks at some point during this year. That is, different individuals participated in the study during different time periods. Consequently, the contexts in which the participants were embedded varied. While the country‐level indicators chosen for the present work are not subject to extreme short‐term fluctuations (e.g. country‐level religiosity does not increase or drop dramatically within months), it is known that perceived disruptions of order, for example, due to terrorist attacks, may activate RWA and its downstream attitudinal consequences (see Osborne et al., 2023). Related to this point, some authors have found evidence of a bidirectional effect between RWA and threat perceptions (Choma & Hodson, 2017; Onraet et al., 2014). The countries included in our global study were subject to different social developments during the period of data collection. For instance, the SARS‐CoV‐2 variant Omicron was first reported at the end of 2021, with different regions subsequently having been affected to different degrees at different times (WHO, 2022). Crucially, the Russian invasion of Ukraine in February 2022 also coincided with the period of data collection. In the following months and years, two of the largest country samples of Study 3, namely Germany and Poland, became the top hosting countries of Ukrainian refugees (UNRIC, 2024). Turkey, as another example of a country with a comparatively large sample size but with a high index in religiosity and societal marginalization, is one of the leading refugee hosts worldwide. With more than 3 million Syrian refugees living in the country and prevailing anti‐refugee sentiments in the political discourse, in 2022, Human Rights Watch documented the deportation of hundreds of Syrians to northern Syria by Turkish officials (Human Rights Watch, 2022). These few examples illustrate the different contextual conditions within which the countries studied where embedded and the need to build on our research by using longitudinal data to assess, first, to which degree country‐level differences are stable or fluctuating and, second, which contextual factors involved have the strongest influence on these differences. Finally, while we have discussed the ways in which RWA, religiosity and (perceived) societal marginalization may affect threat perceptions, including potential interaction effects, we did not actually measure the suggested processes. To do so, future research should combine the current approach with other approaches, such as experimental studies.

Despite our studies' limitations, we hope the present findings will contribute in fruitful ways to the literature on the relationship between RWA and majority groups' perceptions that minoritized groups pose a threat. Future work adopting the current approach of including cross‐sectional and daily diary data with numerous large national samples across the globe and systematically investigating individual‐ and country‐level moderators promises to further refine our knowledge of the link between RWA and threat perceptions.

## AUTHOR CONTRIBUTIONS

**Fahima Farkhari:** Conceptualization; data curation; formal analysis; project administration; resources; visualization; writing – original draft; writing – review and editing. **Julian Scharbert:** Data curation; investigation; methodology; resources; writing – review and editing. **Lara Kroencke:** Formal analysis; investigation; methodology; writing – review and editing. **Christin Schwarzer:** Data curation; formal analysis; visualization; writing – review and editing. **Jonas F. Koch:** Formal analysis; resources; writing – review and editing. **Maarten H. W. van Zalk:** Investigation; methodology; writing – review and editing. **Bernd Schlipphak:** Conceptualization; funding acquisition; investigation; methodology; resources; supervision; writing – review and editing. **Mitja D. Back:** Conceptualization; funding acquisition; investigation; methodology; project administration; resources; supervision; writing – original draft; writing – review and editing.

## CONFLICT OF INTEREST STATEMENT

The authors declare no conflict of interests.

## Supporting information


Data S1.

Data S2.

## Data Availability

We provide our study materials including the preregistration, anonymized data and analysis codes on the OSF (https://osf.io/2excn/).

## Footnotes

^1^
Whereas some coauthors had previously worked with data from the project on which Study 3 relied, the first author was not given access to the data until after the preregistration.

^2^
The reported exclusion criterion was applied as we are interested in the majority group's perceptions that minoritized groups pose a threat.

^3^
In Study 1a, a total of 40 PSM items were presented, as this study was conducted to develop Bollwerk et al.'s (2022) PSM scale. In our analyses, we included only the 15 items from the final scale.

^4^
The differences in the societal context were not least of all reflected in the governmental situations at the time of data collection. The liberal Emmanuel Macron became the president of France in 2017, however, with a substantial support of the political right existent within the country. Germany was governed by the cabinet Merkel III (coalition of the centre‐right and centre‐left) while the right‐wing AfD obtained only a minority of the votes in the preceding federal elections. In Sweden, a Red‐Green minority government was formed, with the support of the Liberals and the Centre Party, however, based on an extensive agreement between the parties and confronted with a strong political right. The Polish government was ruled by the PiS, a right‐wing party which had its best election results in the preceding parliamentary elections and strong support since the first years after its foundation in 2001.

^5^
The assessments were unevenly distributed across countries with some countries providing only a handful of participants. The 10 countries with the most participants were France, Georgia, Germany, Italy, Poland, South Africa, Switzerland, Thailand, Turkey and the United Kingdom with a total sample size of *N* = 2897 after cleaning the data and applying the exclusion criteria. The remaining countries were Argentina, Australia, Austria, Belgium, Brazil, Bulgaria, Cameroon, Canada, Chile, China, Greece, Iran, Israel, Jordan, Lebanon, Luxembourg, Mexico, Morocco, Namibia, Netherlands, Nigeria, Peru, Slovakia, Spain, Tanzania, Uganda, Uruguay, USA, Vietnam, Zambia and Zimbabwe.

^6^
As preregistered, we conducted exploratory analyses using other country‐level measures. These included the following measures: GDP, Hofstede's cultural dimension of individualism versus collectivism, the percentage of migrants in the total population, governmental favouritism of specific religions and governmental populism. Due to the lack of space, these indicators and the results are presented in Appendix C.

^7^
Trait threat represents the mean of symbolic, realistic and safety threats in Studies 1–3.

^8^
The results of the MLMs using daily threat (Study 3) and including all predictors are not directly comparable to the results of the correlational analyses presented in Figure 1, 2, 1‐3. Therefore, regarding daily threat, in addition to the correlation analyses and the MLMs including all predictors, we computed three separate MLMs to predict daily threat based on individual‐level RWA, religiosity or PSM. The results were in line with the correlational findings and comparable to the MLMs based on all three predictors (b_RWA_ = 0.82 [0.74, 0.90]; b_relig_ = 0.12 [0.10, 0.14]; b_PSM_ = 0.46 [0.41, 0.50], all *p* < .001).
