# Supplementary material for: Right‐wing authoritarianism and perceptions that minoritized groups pose a threat: The moderating roles of individual‐ and country‐level religiosity and marginalization
Source: Br J Soc Psychol. 2025 Jan 16;64(1):e12830. doi: 10.1111/bjso.12830 (PMC11737014; doi:10.1111/bjso.12830)
Supplement: Supplementary file 1 — Data S1. [file BJSO-64-0-s002.docx]

**Documentation of the Deviations from the Preregistration**

Most of the deviations from the preregistration originated in the decision to further equalize the procedure of data cleaning and the selection of exclusion criteria across the Studies 1-3. In the following, we report the deviations in detail.

|  | |  |
| --- | --- | --- |
| **Page** | **Exact wording in preregistration** | **Deviation** |
| 2 | “We will request a subset of the data between November 20 and November 27, 2023.” | The first author requested the data on November 25, but then renewed the request on 30 November as in the first data request the variable “education” was not included. |
| 2 | “Our previous studies consisted of cross-sectional survey and computer-assisted telephone interviewing data collected in Germany (Study 1) and in Germany, France, Poland, and Sweden (Study 2; overall *N* = 6,269), respectively. We calculated simple linear regression analyses as well as moderated regression analyses (see Tables A1 and A2 in the Appendix for an overview of the results).” | The overall *N* of Study 1 and 2 after data cleaning and exclusion based on various criteria is now *N* = 5,123 as we decided to equalize the procedure of data cleaning and the selection of exclusion criteria across the studies. However, the results of Study 1 and 2 presented in the preregistration remained largely the same (for a direct comparison compare Tables A1 and A2 of the preregistration (pp. 23-24) with Figure 1 and Table 1 of the main text). |
| 7 | “Therefore, the present study aims to fill this research gap by exploring whether country-level religiosity **(Research Question 4)** and country-level societal marginalization **(Research Question 5)** moderate the association between threat perceptions towards minoritized groups and RWA.” | The wording of this phrase is not entirely accurate. While this should conceptually become very clear from reading the preregistration, it should still be noted that the research questions refer to the moderation of the RWA-Threat link (not the moderation of the Threat-RWA link). |
| 9 | “The original (convenience) sample comprises *N* = 7,490 participants from 71 countries (total ESM measurements = 207,263; total daily measurements = 73,295).^3^ We will exclude all individuals with less than three measurement points regarding daily threat perceptions resulting in *N* = 4,206 participants with 72,182 daily assessments from 56 countries.^4^” | These phrases refer to Study 3. We decided to equalize the procedure of data cleaning and the selection of exclusion criteria across the studies (for details see further below the column referring to page 9/10). After data cleaning and the application of exclusion criteria, *N* = 3,154 participants from 41 countries with *N* = 52,447 total daily measurements remained in the sample. |
| 9 | [Footnote 3]  “The ten countries with most participants were France, Georgia, Germany, Italy, Poland, South Africa, Switzerland, Thailand, Turkey, and the United Kingdom (total *N* of these ten countries = 6,620).” | The total *N* of these ten countries is now lower (*N* = 2,897) due to the additional steps of data cleaning and exclusion as explained further above and further below. |
| 9 | [Footnote 4]  “We will additionally conduct analyses excluding participants who indicated to have a migratory background and/or not the citizenship of the country they reside in as well as participants who indicated a religious affiliation of a minoritized group.” | Mistakenly, it was assumed that the data provided information about the respondents’ religious affiliation while this was not the case. Therefore, the exclusion based on religious affiliation could not be conducted. Moreover, due to the focus of the paper on the majority group’s perception of minoritized groups and for better comparability with the other studies of the present work, the exclusion based on migratory background/foreign citizenship was not conducted in additional analyses but implemented directly. |
| 9/10 | “Data cleaning will be performed as recommended by Scharbert et al. (2023). […] Additionally, all participants whose ESM period lasted longer than 35 days (due to technical issues) will also be excluded.” | As explained further above, additional steps of data cleaning and exclusion were implemented.  The additional exclusion criteria were: non-binary gender (as the sample of those who indicated “other” was too small to consider a third gender category in the analyses); percentage of suspicious ESM data above a specific cut-off value as determined by the authors of the data collection (see section 2.3 of the data paper, Scharbert et al., 2023; this resulted in very few additional exclusions); migratory background; foreign citizenship; either exclusion of respondent’s trait data set or exclusion of respondent’s daily data set after steps for data cleaning and exclusion (i.e., all participants in the final data set should provide both trait and daily level data for comparability of findings). |
| 11/12 | “As a result, 25 countries are considered as low in religiosity (*N* = 2,172), 9 countries are considered as moderate in religiosity (*N* = 1,284), and 22 countries are considered as high in religiosity (*N* = 750; see Table 2).” | Due to the additional steps of data cleaning and the application of additional exclusion criteria, the sample size per category is smaller now and, in some countries (namely, Angola, Czech Republic, Denmark, Ethiopia, Finland, Iceland, India, Ireland, Jamaica, Latvia, Lithuania, Norway, Portugal, Sweden, UAE), no participants are left in the final sample anymore.  As a result, 21 countries were considered low (N = 1,500), six countries were considered moderate (N = 1,041), and 14 countries high in religiosity (N = 613; see Table A1 in Appendix A for individual indices for each country) |
| 12 | Table 2 | Table A1 in the Appendix A presents the updated version of Table 2 including the updated number of participants for each country after the additional steps of data cleaning and the application of the additional exclusion criteria. |
| 15 | “As a result, 22 countries are considered as low (N = 2,780), 18 countries as moderate (N = 990), and 16 countries as high in marginalization (N = 436; see Table S1 in the Online Appendix for details).” | First of all, that phrase includes a typo as it says that 9 countries are moderate in marginalization while it is actually 18 countries. Table 2 of the preregistration correctly indicates 18 countries to be in the category of moderate marginalization.  As a result, 19 countries were considered low (N = 2,029), 14 countries moderate (N = 754), and eight countries high in marginalization (N = 371; see Table A1 in Appendix A for individual indices for each country).” |
| 15 | **[in the section: Analyses using cross-sectional measurements of threat perceptions]** “To answer our second set of research questions (including RQ2 and RQ3; see number 2a) in Table 1), threat perceptions will be predicted by individual-level RWA, including individual-level religiosity or individual-level PSM as moderator variables (two separate models). […] Continuous individual-level predictors (RWA, religiosity, PSM) will be centered at the sample mean prior to the analyses. […]”  **[in the section: Analyses using cross-sectional measurements of threat perceptions]** “To answer our third set of research questions (including RQ4 and RQ5; see number 2b) in Table 1), (…). Continuous individual-level predictors (RWA, religiosity, PSM) will be centered at the sample mean prior to the analyses.”  **[in the section: Analyses using daily measurements of threat perceptions]** “To answer our third set of research questions (including RQ4 and RQ5), we will conduct multilevel analyses implementing individual-level RWA as level-2 predictor, the dummy-coded country-level religiosity and societal marginalization variables as level-2 moderators, and daily threat perceptions as level-1 outcome.” | For better comparability with the results of Study 1 and 2, which used trait threat measures, we did not apply mean-centering in the analyses using the trait threat measure of Study 3. Instead, we applied z-standardization. We only applied mean-centering when conducting multilevel modelling using the daily threat measure of Study 3. |
| 16 | “Specifically, we will calculate the regression coefficients for the prediction of threat perceptions by RWA individually for each country with *N* > 50 respondents, using traditional simple linear regression analyses. This applies to 13 countries of which six (seven) are low, three (three) moderate and four (three) high in religiosity (societal marginalization; see Table 3).” | Australia (N = 26), China (N = 44) and USA (N = 37) had N < 50 respondents after exclusion of respondents based on foreign citizenship and migrant background. These countries were therefore not included in the supplementary analyses. Consequently, 10 countries were left of which four (five) are low, two (three) moderate and four (two) high in religiosity (societal marginalization; see Table C1 in Appendix C). |

In addition, we preregistered for Study 3 to conduct all analyses using the separate RWA facets, using the separate PSM facets, and using the separate threat facets. Due to lack of space, we refrained from doing so. However, the threat facets were highly correlated with each other. Accordingly, RWA was similarly related to the different facets of trait threat (*r_symb_* = .47, *r_real_* = .49, *r_safe_* = .46; the daily threat measure did not differentiate between types of threat). The correlation of the RWA subdimensions with threat perceptions differed to some extent, but the differences were not dramatic (*r* = .34 ≤ *r* ≤.46 for trait threat; *r* = .23 ≤ *r* ≤.33 for average daily threat). The correlations between the PSM subdimensions and threat perceptions differed more for trait threat (*r* = .26 ≤ *r* ≤.43) and less for average daily threat (*r* = .22 ≤ *r* ≤.33).
