# Supplementary material for: Right‐wing authoritarianism and perceptions that minoritized groups pose a threat: The moderating roles of individual‐ and country‐level religiosity and marginalization
Source: Br J Soc Psychol. 2025 Jan 16;64(1):e12830. doi: 10.1111/bjso.12830 (PMC11737014; doi:10.1111/bjso.12830)
Supplement: Supplementary file 2 — Data S2. [file BJSO-64-0-s001.docx]

**Appendix A**

**A1. Data cleaning**

**Data cleaning of Study 1**

Study 1a

As the survey was prolonged once, the original data set consists of two data sets (*N_1_* = 1258; *N_2_* = 990) which have been merged into one data set (*N_total_* = 2,248). However, *N* = 1,245 participants did not give their consent for using their data. Accordingly, we consider *N* = 1,003 as the original sample prior to data cleaning. Participants were excluded based on the following criteria: non-binary gender (as the sample of those who indicated “other” was too small to consider a third gender category in the analyses); non-Christian affiliation (i.e., Islam, Judaism, other non-Christian religious affiliation); non-German citizenship; lack of variance in responses (no variance in any of the scales measuring RWA, PSM, and threat); on average lower response time than 2 seconds per item^[[1]](#footnote-1)^.

No exclusion was made based on missing values as the panel provider did not permit missing data.

Study 1b

The original data set consists of *N* = 1,023 participants. Participants who did not give their consent for using their data or who had missing values had previously been excluded from the data. We excluded participants based on the following criteria: non-Christian affiliation (Islam, Judaism, other non-Christian religious affiliation); non-German citizenship; indicators of careless responding; lack of variance in responses (no variance in any of the scales measuring RWA, PSM, and threat).

No respondent was excluded based on non-binary gender as nobody indicated “diverse” as their gender. No respondent was excluded based on response time as nobody had a lower average response time than 2 seconds per item.

**Data cleaning of Study 2**

The original data set consists of *N* = 5,011 participants. Participants who did not give their consent for using their data had previously already been excluded from the data. Participants were excluded based on the following criteria: non-Christian affiliation (Islam, Judaism, other non-Christian religious affiliation); migratory background (self or parents); lack of variance in responses (no variance in any of the scales measuring RWA, PSM, and threat); missing values (as the central variables of our study were measured with either single items or very short scales, participants with any missing values were excluded).

In Study 2, there no exclusion was made based on non-binary gender as the survey did not offer the option to indicate non-binary gender. Furthermore, no exclusion was made based on response times as the survey was conducted via computer-assisted telephone interviewing.

**Data cleaning of Study 3**

The original sample of Study 3 comprised N = 7,490 participants from 71 countries (total daily measurements = 73,295).

Data cleaning was performed according to the recommendations of Scharbert et al. (2023). It was based on indications of carelessness found in the trait-level and daily-level surveys as suggested in previous recommendations (Curran, 2016; Meade & Craig, 2012; Geerarts & Kuppens, 2020; Ward & Meade, 2023). In the following, the procedure for data cleaning regarding the trait and daily surveys is described separately.

*Trait-level data cleaning*

Data cleaning in the trait-level surveys was conducted according to the following criteria: a) respondent indicated in the T2-survey that he/she did not answer the surveys conscientiously; b) unrealistic average response times (average response time of < 2 seconds per item); c) identical responses to all items (e.g., 1-1-1-1-1-1) in at least one of two questionnaires which were presented at the beginning and in the middle of the trait survey, respectively, and which were answered in a matrix-like Likert-Scale scheme which makes them prone to careless responding; or a diagonal pattern of response (e.g., 1-2-3-4-5-6-5-4-3-2-1) regarding at least half of the items in at least one of those two questionnaires; d) strong agreement to opposing items (e.g., happy – sad).

*Daily-level data cleaning*

Data cleaning in the daily-level surveys was conducted according to the following criteria: a) unrealistic average response times in state surveys (average response time of < 1.5 seconds per item; the cut-off value is lower compared to the trait-level data cleaning due to increasing familiarity following the daily repetition); b) identical responses to all items or a diagonal pattern of response regarding the items on prejudice following the same logic as described for the trait-level surveys; c) unrealistic time indication regarding the time of falling asleep (outside of the time period between 7pm and 7am) or the time of waking up (outside the time period between 4am and 2pm).

Furthermore, we excluded participants based on the following criteria: non-binary gender (as the sample of those who indicated “other” was too small to consider a third gender category in the analyses); indication to have a migratory background or not the citizenship of the respective country; less than three measurement points regarding daily threat perceptions; percentage of suspicious ESM data above a specific cut-off value as determined by the authors of the data collection (see section 2.3 of the data paper, Scharbert et al., 2022; this resulted in very few additional exclusions); either exclusion of respondent’s trait data set or exclusion of respondent’s daily data set after application of the above-mentioned steps (i.e., all participants in the final data set should provide both trait and daily level data for comparability of findings).

**A2. Measures**

**Measure of Muslim threat (self-generated items based on the refugee threat scale of Landmann et al., 2019) in Study 2**

The values and beliefs of Muslims are incompatible with the general values and beliefs in Germany.

The costs of integration programs for Muslims are a burden on Germany.

Muslims living here are a threat to security in Germany.

Muslims threaten our way of life and our values in Germany.

Muslims threaten the economic situation in Germany.

There are more acts of violence in Germany because of Muslims.

**Measure of refugee threat (shortened versions of the items of the refugee threat scale of Landmann et al., 2019) in Study 2**

The values and beliefs of refugees are not compatible with those of Germans.

Refugees threaten the economic situation in Germany.

Refugees threaten security in Germany.

**Measure of trait threat in Study 3**

Trait-level threat perceptions were measured with six items introduced by the instruction “The following statements refer to people in your country who belong to a different ethnic, religious, or national group than yourself. For each statement, please indicate the extent to which you agree with that statement”.

These people threaten the way of life and the values in our country.

The values and beliefs of these people are incompatible with those of people in our country.

The costs of integration programs for these people put a strain on our country.

These people threaten the economic situation in our country.

These people threaten the public safety in our country.

Because of these people, there are more acts of violence in our country.

**Country-level measures (additional information on indices of marginalization)**

The Gini index (income distribution) measures the degree to which the distribution of income among individuals or households is distributed equally within a country (0% = perfect equality; 100% = maximum inequality).

The ratings of civil liberties as reported in the “Freedom in the World” are published in an annual global report on the current state of political rights and civil liberties in 195 countries. Expert consultants from academia, think tanks, human rights organizations, and internal and external analysts draw on various sources (e.g., news articles, academic analyses, reports from NGOs) to assess a country’s civil liberties across four subcategories (freedom of expression and belief; associational and organizational rights; rule of law; personal autonomy and individual rights). A total of 15 indicators are assigned 0 to 4 points each, so that the final score can range from 0 to 60 per country.

The Democracy Index provides an overview of the state of democracy in 165 countries. It results from expert assessments and data of public opinion surveys (e.g., World Values Survey and Eurobarometer) on 60 indicators across five categories. The category political participation is covered by nine indicators (see list further below).

**Country-level measures (items/criteria/indicators)**

**Criteria for the rating of civil liberties taken from Freedom in the World:**

Freedom of expression and belief

- Are there free and independent media?
- Are individuals free to practice and express their religious faith or nonbelief in public and private?
- Is there academic freedom, and is the educational system free from extensive political indoctrination?
- Are individuals free to express their personal views on political or other sensitive topics without fear of surveillance or retribution?

Associational and organizational rights

- Is there freedom of assembly?
- Is there freedom for nongovernmental organizations, particularly those that are engaged in human rights– and governance-related work?
- Is there freedom for trade unions and similar professional or labor organizations?

Rule of law

- Is there an independent judiciary?
- Does due process prevail in civil and criminal matters?
- Is there protection from the illegitimate use of physical force and freedom from war and insurgencies?
- Do laws, policies, and practices guarantee equal treatment of various segments of the population?

Personal autonomy and individual rights

- Do individuals enjoy freedom of movement, including the ability to change their place of residence, employment, or education?
- Are individuals able to exercise the right to own property and establish private businesses without undue interference from state or nonstate actors?
- Do individuals enjoy personal social freedoms, including choice of marriage partner and size of family, protection from domestic violence, and control over appearance?
- Do individuals enjoy equality of opportunity and freedom from economic exploitation?

**Indicators of the category “Political participation” from the Democracy Index:**

- Voter participation/turnout for national elections. [1 if above 70%; 0.5 if 50%-70%; 0 if below 50% // If voting is obligatory, score 0. Score 0 if scores for questions 1 or 2 is 0]
- Do ethnic, religious, and other minoritized groups have a reasonable degree of autonomy and voice in the political process? [1: Yes; 0.5: Yes, but serious flaws exist; 0: No]
- Women in parliament. [1 if more than 20% of seats; 0.5 if 10-20%; 0 if less than 10%]
- Extent of political participation. Membership of political parties and political non-governmental organizations. [Score 1 if over 7% of population for either; Score 0.5 if 4-7%.; Score 0 if under 4%. // If participation is forced, score 0]
- Citizens’ engagement with politics. [1: High; 0.5: Moderate; 0: Low // If available, from World Values Survey, percentage of people who are very or somewhat interested in politics: 1 if over 60%; 0.5 if 40-60%; 0 if less than 40%]
- The preparedness of population to take part in lawful demonstrations. [1: High; 0.5: Moderate; 0: Low // If available, from World Values Survey, percentage of people who have taken part in or would consider attending lawful demonstrations: 1 if over 40%; 0.5 if 30-40%; 0 if less than 30%]
- Adult literacy. [1 if over 90%; 0.5 if 70-90%; 0 if less than 70%]
- Extent to which adult population shows an interest in and follows politics in the news. [1: High; 0.5: Moderate; 0: Low // If available, from World Values Survey, percentage of population that follows politics in the news media (print, TV, or radio) every day: 1 if over 50%; 0.5 if 30-50%; 0 if less than 30%]
- The authorities make a serious effort to promote political participation. [1: Yes; 0.5: Some attempts; 0: No // Consider the role of the education system, and other promotional efforts. Consider measures to facilitate voting by members of the diaspora. If participation is forced, score 0.]

**A3. Overview of countries and their individual religiosity and marginalization indices**

**Table A1**

*Country-level indices of religiosity and marginalization.*

| Country |  | | Religiosity | | | |  | Marginalization | | | | |
| --- | --- | --- | --- | --- | --- | --- | --- | --- | --- | --- | --- | --- |
|  | *N* | | Index | | | Category |  | Economic | Cultural | Political | Mean | Category |
| Argentina | 32 | | 63,4 | | | moderate |  | 42,3 | 18,3 | 27,8 | 29,5 | moderate |
| Australia | 26 | | 32,3 | | | low |  | 34,3 | 6,7 | 22,2 | 21,1 | low |
| Austria | 3 | | 46,4 | | | low |  | 30,2 | 6,7 | 11,1 | 16 | low |
| Belgium | 13 | | 33,5 | | | low |  | 27,2 | 5 | 50 | 27,4 | moderate |
| Brazil | 20 | | 89,4 | | | high |  | 48,9 | 30 | 38,9 | 39,3 | moderate |
| Bulgaria | 2 | | 39,4 | | | low |  | 40,3 | 23,3 | 27,8 | 30,5 | moderate |
| Cameroon | 1 | | 94 | | | high |  | 46,6 | 85 | 61,1 | 64,2 | high |
| Canada | 5 | | 41,4 | | | low |  | 33,3 | 3,3 | 11,1 | 15,9 | low |
| Chile | 1 | | 68 | | | moderate |  | 44,9 | 6,7 | 44,4 | 32 | moderate |
| China | 44 | | 15,8 | | | low |  | 38,2 | 81,7 | 72,2 | 64 | high |
| France | 315 | | 28,8 | | | low |  | 32,4 | 15 | 22,2 | 23,2 | low |
| Georgia | 141 | | 80,5 | | | high |  | 34,5 | 40 | 44,4 | 39,6 | moderate |
| Germany | 887 | | 39,5 | | | low |  | 31,7 | 8,3 | 16,7 | 18,9 | low |
| Greece | 1 | | 71,3 | | | moderate |  | 33,1 | 16,7 | 38,9 | 29,6 | moderate |
| Iran | 13 | | 82,5 | | | high |  | 40,9 | 83,3 | 61,1 | 61,8 | high |
| Israel | 14 | | 45 | | | low |  | 38,6 | 30 | 0 | 22,9 | low |
| Italy | 553 | | 65,2 | | | moderate |  | 35,2 | 10 | 27,8 | 24,3 | low |
| Jordan | 1 | | 94,4 | | | high |  | 33,7 | 63,3 | 61,1 | 52,7 | high |
| Lebanon | 2 | | 85,2 | | | high |  | 31,8 | 51,7 | 33,3 | 38,9 | moderate |
| Luxembourg | 2 | | 35,3 | | | low |  | 34,2 | 1,7 | 33,3 | 23,1 | low |
| Mexico | 8 | | 63,3 | | | moderate |  | 45,4 | 45 | 27,8 | 39,4 | moderate |
| Morocco | 1 | | 94,7 | | | high |  | 39,5 | 60 | 44,4 | 48 | high |
| Namibia | 2 | | 90,8 | | | high |  | 59,1 | 23,3 | 33,3 | 38,6 | moderate |
| Netherlands | 11 | | 30,6 | | | low |  | 29,2 | 3,3 | 16,7 | 16,4 | low |
| Nigeria | 3 | | 95,1 | | | high |  | 35,1 | 61,7 | 61,1 | 52,6 | high |
| Peru | 3 | | 83,3 | | | high |  | 43,8 | 30 | 44,4 | 39,4 | moderate |
| Poland | 409 | | 66,9 | | | moderate |  | 30,2 | 21,7 | 33,3 | 28,4 | moderate |
| Slovakia | 1 | | 47,7 | | | low |  | 23,2 | 11,7 | 44,4 | 26,4 | moderate |
| South Africa | 119 | | 85,1 | | | high |  | 63 | 23,3 | 16,7 | 34,3 | moderate |
| Spain | 2 | | 39 | | | low |  | 34,3 | 11,7 | 27,8 | 24,6 | low |
| Switzerland | 76 | | 40,8 | | | low |  | 33,1 | 5 | 22,2 | 20,1 | low |
| Tanzania | 1 | | 96 | | | high |  | 40,5 | 63,3 | 50 | 51,3 | high |
| Thailand | 88 | | 95,7 | | | high |  | 35 | 60 | 33,3 | 42,8 | high |
| Turkey | 213 | | 79,7 | | | high |  | 41,9 | 73,3 | 44,4 | 53,2 | high |
| Uganda | 1 | | 94,9 | | | high |  | 42,7 | 61,7 | 61,1 | 55,2 | high |
| UK | 96 | | 29,9 | | | low |  | 35,1 | 10 | 16,7 | 20,6 | low |
| Uruguay | 2 | | 42,6 | | | low |  | 40,2 | 5 | 27,8 | 24,3 | low |
| USA | 37 | | 65 | | | moderate |  | 41,5 | 15 | 11,1 | 22,5 | low |
| Vietnam | 1 | | 34 | | | low |  | 35,7 | 73,3 | 61,1 | 56,7 | high |
| Zambia | 2 | | 94,8 | | | high |  | 57,1 | 50 | 50 | 52,4 | high |
| Zimbabwe | 2 | | 88,8 | | | high |  | 50,3 | 71,7 | 61,1 | 61 | high |
|  |  | |  | | |  |  |  |  |  |  |  |
|  | |  | |  |  | | |  |  |  |  |  |

*Note.* UAE = United Arab Emirates; UK = United Kingdom. *N* = 1,500 (*N* = 2,029) respondents are of countries categorized as low in religiosity (marginalization), *N* = 1,041 (*N* = 754) respondents are of countries categorized as moderate in religiosity (marginalization), *N* = 613 (*N* = 371) respondents are of countries categorized as high in religiosity (marginalization).

**References**

Curran, P. G. (2016). Methods for the detection of carelessly invalid responses in survey data. *Journal of Experimental Social Psychology*, *66*, 4–19. <https://doi.org/10.1016/j.jesp.2015.07.006>

Geeraerts, J., & Kuppens, P. (2020). *Investigating careless responding detection techniques in experience sampling methods (ESM) [PDF slides].* Research Group of Quantitative Psychology and Individual Difference, KU Leuven. <https://osf.io/8ymh5>

Meade, A. W., & Craig, S. B. (2012). Identifying careless responses in survey data. *Psychological Methods*, *17*(3), 437–455. <https://doi.org/10.1037/a0028085>

Ward, M. K., & Meade, A. W. (2023). Dealing with careless responding in survey data: Prevention, identification, and recommended best practices. *Annual Review of Psychology*, *74*, 577-596. <https://doi.org/10.1146/annurev-psych-040422-045007>

**Appendix B**

**Table B1**

*Descriptive Statistics of the Final Samples*

|  | Study 1a | | Study 1b | |  | Study 2 | | | | | | | |  | Study 3 | |
| --- | --- | --- | --- | --- | --- | --- | --- | --- | --- | --- | --- | --- | --- | --- | --- | --- |
| Variable | Germany | | Germany | |  | France | | Germany | | Poland | | Sweden | |  | *N* = 41 countries | |
|  | *M* | SD | *M* | SD |  | *M* | SD | *M* | SD | *M* | SD | *M* | SD |  | *M* | SD |
| Threat Perceptions | 2.33 | 1.07 | 3.25 | 1.18 |  | 2.91 | 1.36 | 2.51 | 1.16 | 2.64 | 1.27 | 2.84 | 1.33 |  | 2.16  (2.68) | 1.16  (1.79) |
| Age | 45.36 | 16.25 | 47.35 | 15.48 |  | 59.42 | 16.22 | 53.37 | 15.62 | 48.75 | 15.99 | 57.48 | 16.83 |  | 30.52 | 13.95 |
| Female | .61 | .49 | 0.50 | 0.50 |  | 0.50 | 0.50 | 0.45 | 0.50 | 0.45 | 0.50 | 0.41 | 0.49 |  | 0.79 | 0.41 |
| Education | 7.39 | 1.87 | 5.14 | 2.15 |  | 3.98 | 2.16 | 4.48 | 2.12 | 5.02 | 1.94 | 4.35 | 1.74 |  | 4.67 | 1.01 |
| RWA | 2.23 | 0.74 | 3.09 | 0.78 |  | 4.65 | 1.12 | 3.57 | 1.11 | 4.20 | 1.16 | 3.32 | 1.10 |  | 2.44 | 0.75 |
| Religiosity | 2.63 | 1.73 | 2.65 | 1.70 |  | 2.43 | 1.48 | 2.62 | 1.48 | 3.43 | 1.60 | 2.25 | 1.33 |  | 3.95 | 2.94 |
| PSM | 3.23 | 1.15 | 3.96 | 1.09 |  | 3.96 | 1.26 | 3.28 | 1.20 | 4.06 | 1.21 | 2.99 | 1.13 |  | 3.55 | 1.25 |
| [.36, .48] |  |  |  | [.35, .46] |  | [.26, .38] | [.20, .33] | [.13, .26] | [−.03, .10] | [−.07, .05] | [.39, .52] | [.28, .41] |  | [.39, .52] |  |  |
| *N* | 923 | | 973 | |  | 723 | | 940 | | 865 | | 699 | |  | 3154 | |

*Note. M* = Mean; SD = Standard deviation; *RWA* = Right-Wing Authoritarianism. *PSM* = Perceived Societal Marginalization. *N* = total sample size. Gender was dummy-coded (0 = male, 1 = female). Education ranges from 0 (no degree) to 10 (PhD) in Study 1a and Study 1b. In Study 2, the educational levels in the respective countries were transformed into ISCED levels (0 to 8). In Study 3, education ranges from 1 (no qualification) to 7 (doctorate or PhD). In Study 1a and Study 1b, threat perceptions towards refugees were measured. In Study 2, threat perceptions towards refugees and Muslims were measured and aggregated. In Study 3, threat perceptions towards people in one’s own country who belong to a different ethnic, religious, or national group than oneself were measured. The values in brackets refer to the mean and standard deviation of average daily threat perceptions in Study 3.

**Table B2**

*Multiple Linear Regression Models and Multilevel Models Predicting Threat Perceptions (Including RWA, Religiosity, and PSM)*

|  |  | Study 1 | | Study 2 | | | | Study 3 (Trait threat) | Study 3 (Daily threat) |  |  |
| --- | --- | --- | --- | --- | --- | --- | --- | --- | --- | --- | --- |
| Variable |  | Germany | Germany | France | Germany | Poland | Sweden | N = 41 countries | N= 41 countries |  |  |
|  |  | β | β | β | β | β | β | β | b |  |  |
|  |  |  |  |  |  |  |  |  |  |  |  |
| RWA |  | **.52** | **.40** | **.30** | **.33** | **.22** | **.31** | **.40** | **0.59** |  |  |
|  |  | [.46, .57] | [.35, .46] | [.23, .36] | [.27, .38] | [.16, .28] | [.24, .37] | [.37, .43] | [0.50, 0.67] |  |  |
| Religiosity |  | −.05 | **−.09** | **.16** | .00 | **.33** | .03 | **.10** | **0.05** |  |  |
|  |  | [−.09, .00] | [−.15, −.04] | [.10, .23] | [−.05, .05] | [.27, .39] | [−.03, .09] | [.07, .13] | [0.03, 0.07] |  |  |
| PSM |  | **.25** | **.30** | **.24** | **.43** | **.07** | **.43** | **.25** | **0.33** |  |  |
|  |  | [.20, .31] | [.24, .35] | [.17, .31] | [.37, .48] | [.01, .13] | [.37, .49] | [.22, .28] | [0.28, 0.38] |  |  |
|  |  |  |  |  |  |  |  |  |  |  |  |
| *N* |  | 923 | 973 | 723 | 940 | 865 | 699 | 3,154 | 3,154 |  |  |
|  |  |  |  |  |  |  |  |  |  |  |  |

*Note.* *RWA* = Right-Wing Authoritarianism. *PSM* = Perceived Societal Marginalization. *N* = total sample size*.* Bold numbers represent significant coefficients (*p* < .05). Coefficients represent coefficients of MLR for Study 1, 2 and 3 (trait threat; β) and coefficients of MLM for Study 3 (daily threat; b). Continuous predictors were *z*-standardized prior to the analyses using MLR and mean-centered prior to the analyses using MLM. Gender was dummy-coded (0 = male, 1 = female). Education ranges from 0 (no degree) to 10 (PhD) in Study 1a and Study 1b. In Study 2, the educational levels in the respective countries were transformed into ISCED levels (0 to 8). In Study 3, education ranges from 1 (no qualification) to 7 (doctorate or PhD). In Study 1a and Study 1b, threat perceptions towards refugees were measured. In Study 2, threat perceptions towards refugees and Muslims were measured and aggregated. In Study 3, threat perceptions towards people in one’s own country who belong to a different ethnic, religious, or national group than oneself were measured.

**Table B3**

*Multiple Linear Regression Models and Multilevel Models Predicting Threat Perceptions (Including RWA, Religiosity, PSM, and Control Variables)*

|  | Study 1 | |  | Study 2 | | | |  | Study 3 | |
| --- | --- | --- | --- | --- | --- | --- | --- | --- | --- | --- |
|  |  |  |  |  |  |  |  |  | Trait threat | Daily threat |
|  | Germany | Germany |  | France | Germany | Poland | Sweden |  | *N* = 41 countries | *N* = 41 countries |
|  | β | β |  | β | β | β | β |  | β | b (MLM) |
|  |  |  |  |  |  |  |  |  |  |  |
| Age | .05 | −.01 |  | **.21** | **.13** | −.06 | .05 |  | **.07** | −0.07 |
|  | [.00, .10] | [−.06, .05] |  | [.14, .28] | [.07, .18] | [−.12, .00] | [−.01, .11] |  | [.04, .10] | [−0.21, 0.08] |
| Gender | **−.08** | .02 |  | −.02 | −.02 | .01 | **−.07** |  | **−.02** | 0.00 |
|  | [−.13, −.03] | [−.03, .07] |  | [−.08, .04] | [−.07, .03] | [−.05, .07] | [−.13, −.01] |  | [−.05, −.00] | [−0.00, 0.01] |
| Education | .03 | **−.07** |  | **−.13** | **−.06** | **−.16** | **−.06** |  | −.00 | 0.06 |
|  | [−.02, .08] | [−.12, −.01] |  | [−.20, −.06] | [−.02, −.01] | [−.22, −.10] | [−.00, −.13] |  | [−.03, .03] | [−0.00, 0.12] |
| RWA | **.52** | **.39** |  | **.24** | **.30** | **.23** | **.28** |  | **.39** | **0.59** |
|  | [.46, .57] | [.33, .45] |  | [.18, .31] | [.24, .35] | [.17, .29] | [.22, .35] |  | [.36, .42] | [0.50, 0.67] |
| Religiosity | **−.05** | **−.09** |  | **.12** | −.01 | **.31** | .04 |  | **.10** | **0.05** |
|  | [−.10, −.00] | [−.15, −.04] |  | [.06, .19] | [−.07, .04] | [.25, .37] | [−.02, .10] |  | [.07, .13] | [0.03, 0.07] |
| PSM | **.25** | **.29** |  | **.22** | **.40** | .05 | **.40** |  | **.25** | **0.33** |
|  | [.20, .31] | [.23, .34] |  | [.16, .291] | [.34, .45] | [−.02, .11] | [.34, .47] |  | [.22, .28] | [0.28, 0.38] |
|  |  |  |  |  |  |  |  |  |  |  |
| *N* | 923 | 973 |  | 723 | 940 | 865 | 699 |  | 3,154 | 3,154 |

*Note. RWA* = Right-Wing Authoritarianism. *PSM* = Perceived Societal Marginalization. *N* = total sample size*.* Bold numbers represent significant coefficients (*p* < .05). Coefficients represent coefficients of MLR for Study 1, 2 and 3 (trait threat) and coefficients of MLM for Study 3 (daily threat). All models included RWA, religiosity, PSM, and socio-demographic control variables. Continuous predictors were *z*-standardized prior to the analyses using MLR and mean-centered prior to analyses using MLM. Gender was dummy-coded (0 = *male*, 1 = *female*). Education ranges from 0 (*no degree*) to 10 (*PhD*) in Study 1a and Study 1b. In Study 2, the educational levels in the respective countries were transformed into ISCED levels (0 to 8). In Study 3, education ranges from 1 (no qualification) to 7 (doctorate or PhD). In Study 1a and Study 1b, threat perceptions towards refugees were measured. In Study 2, threat perceptions towards refugees and Muslims were measured and aggregated into one measure. In Study 3, threat perceptions towards people in one’s own country who belong to a different ethnic, religious, or national group than oneself were measured.

**Table B4**

*Simple Slopes Models (Moderating Effects of Individual-Level Religiosity or Perceived Societal Marginalization on the RWA-Threat Link)*

|  | Study 1 | |  | Study 2 | | | | Study 3 | |
| --- | --- | --- | --- | --- | --- | --- | --- | --- | --- |
|  |  |  |  |  |  |  |  | Trait threat | Daily threat |
|  | Germany | Germany |  | France | Germany | Poland | Sweden | N = 41 countries | N = 41 countries |
| Predictor | β | β |  | β | β | b | β | β | b |
|  |  |  |  |  |  |  |  |  |  |
| **Individual-Level Religiosity** |  |  |  |  |  |  |  |  |  |
| −1 SD | −.03 | −.08 |  | **.21** | .04 | **.23** | .05 | **.56** | **0.70** |
|  | [−.00, .05] | [−.16, −.01] |  | [.10, .32] | [−.04, .12] | [.15, .31] | [−.06, .16] | [.50, .62] | [0.58, 0.82] |
| +1 SD | −.06 | **−.14** |  | **.21** | .00 | **.40** | .09 | **.65** | **0.79** |
|  | [−.13, .01] | [−.22, .07] |  | [.11, .31] | [−.07, .07] | [.32, .48] | [−.02, .21] | [.59, .70] | [0.68, 0.90] |
|  |  |  |  |  |  |  |  |  |  |
|  |  |  |  |  |  |  |  |  |  |
| **Individual-Level PSM** |  |  |  |  |  |  |  |  |  |
| −1 SD | **.13** | **.33** |  | **.22** | **.40** | **.10** | **.44** | **.30** | **0.50** |
|  | [.06, .20] | [.26, .40] |  | [.12, .32] | [.33, .47] | [.00, .19] | [.34, .54] | [.26, .34] | [0.38, 0.62] |
| +1 SD | **.36** | **.27** |  | **.34** | **.43** | **.**06 | **.58** | **.48** | **0.75** |
|  | [.29, .43] | [.20, .35] |  | [.24, .45] | [.36, .50] | [−.03, .15] | [.48, .68] | [.44, .52] | [0.65, 0.85] |
|  |  |  |  |  |  |  |  |  |  |

*Notes.* *RWA* = Right-Wing Authoritarianism. *PSM* = Perceived Societal Marginalization. *N* = total sample size*.* Bold numbers represent significant coefficients (*p* < .05). The coefficients represent coefficients from the moderated multiple linear regression analyses in Studies 1, 2, and 3 (trait threat); RWA, religiosity, PSM, and threat perceptions were *z*-standardized prior to the analyses. The coefficients represent coefficients from moderated multilevel modeling for Study 3 (daily threat); RWA, religiosity, and PSM were mean-centered prior to the analyses. In Studies 1a and 1b, threat perceptions were measured with respect to refugees. In Study 2, threat perceptions were measured with respect to refugees and Muslims and aggregated into one measure. In Study 3, threat perceptions were measured with respect to people in one’s own country who belong to a different ethnic, religious, or national group than oneself.

**Table B5**

*Moderating Effects of Country-Level Religiosity or Marginalization on the RWA-Threat Link (Study 3).*

|  | Trait threat | | Daily threat | |
| --- | --- | --- | --- | --- |
|  | β | β | *b* | *b* |
|  |  |  |  |  |
| RWA (model with ref = low for country variables) | **.40**  [.35, .44] | **.42**  [.38, .46] | **0.67**  [0.54, 0.79] | **0.60**  [0.49, 0.70] |
| RWA (model with ref = moderate for country variables) | **.51**  [.46, .56] | **.49**  [.44, .55] | **0.85**  [0.72, 0.99] | **0.71**  [0.55, 0.86] |
|  |  |  |  |  |
| Country relig (high vs. low) | **.53**  [.46, .60] |  | **1.06**  [0.89, 1.22] |  |
| Country relig (high vs. moderate) | **.45** |  | **0.49** |  |
|  | [.38, .53] |  | [0.32, 0.66] |  |
| Country relig (moderate vs. low) | **.08**  **[**.02, .14] |  | **0.57**  [0.43, 0.71] |  |
|  |  |  |  |  |
|  |  |  |  |  |
| Country marg. (high vs. low) |  | **.85**  [.76, .93] |  | **1.57**  [1.38, 1.76] |
| Country marg. (high vs. moderate) |  | **.72**  [.63, .82] |  | **0.73**  [0.52, 0.95] |
| Country marg. (moderate vs. low) |  | **.12** |  | **0.84** |
|  |  | [.06, .19] |  | [0.69, 0.98] |
|  |  |  |  |  |
| RWA*Country relig (high vs. low) | **−.09**  [−.16, −.01] |  | −**0.43**  [−0.65, −0.21] |  |
| RWA*Country relig (high vs. moderate) | **−.20**  [−.38, −.12] |  | −**0.61**  [−0.83, −0.39] |  |
| RWA*Country relig (moderate vs. low) | **.11**  [.05, .18] |  | **0.18**  [0.00, 0.37] |  |
|  |  |  |  |  |
| RWA*Country marg. (high vs. low) |  | **−.28**  [−.37, −.20] |  | −**0.54**  [−0.79, −0.30] |
| RWA*Country marg. (high vs. moderate) |  | **−.36**  [−.45, −.26] |  | −**0.65**  [−0.92, −0.38] |
| RWA*Country marg. (moderate vs. low) |  | **.08**  [.01, .14] |  | 0.11  [−0.08, 0.30] |

*Note. RWA* = Right-Wing Authoritarianism. *PSM* = Perceived Societal Marginalization. *N* = 3,154. Bold numbers represent significant coefficients (*p* < .05). Coefficients stem from moderated MLR using trait threat (RWA and threat z-standardized prior to the analyses) and from moderated MLM when using daily threat (RWA mean-centered prior to the analyses).

**Appendix C**

**C1. Results of preregistered supplementary analyses.**

In the following, we begin with presenting the results of the preregistered supplementary analyses which we applied to examine the moderating role of country-level religiosity and marginalization in the RWA-Threat link. For Study 3, we pre-registered and conducted two supplementary analyses including only the 10 countries with N > 50 respondents (see Table C1).

**Table C1**

*Country-Level Religiosity and Marginalization of Countries with N > 50 (Study 3).*

| Country-Level Variables | | | | | | | | |
| --- | --- | --- | --- | --- | --- | --- | --- | --- |
|  | | | |  |  | | | |
| **Country-Level Religiosity** | | | |  | **Country-Level Marginalization** | | | |
|  |  |  |  |  |  |  |  |  |
|  | Country | *N* | Index |  |  | Country | *N* | Index |
|  |  |  |  |  |  |  |  |  |
| **high** | Georgia | 141 | 80.5 |  | **high** | Thailand | 88 | 42.8 |
|  | South Africa | 119 | 85.1 |  |  | Turkey | 213 | 53.2 |
|  | Thailand | 88 | 95.7 |  |  |  |  |  |
|  | Turkey | 213 | 79.7 |  |  |  |  |  |
|  |  |  |  |  |  |  |  |  |
| **moderate** | Italy | 553 | 65.2 |  | **moderate** | Georgia | 141 | 39.6 |
|  | Poland | 409 | 66.9 |  |  | Poland | 409 | 28.4 |
|  |  |  |  |  |  | South Africa | 119 | 34.3 |
|  |  |  |  |  |  |  |  |  |
| **low** | France | 315 | 28.8 |  | **low** | France | 315 | 23.2 |
|  | Germany | 887 | 39.5 |  |  | Germany | 887 | 18.9 |
|  | Switzerland | 76 | 40.8 |  |  | Italy | 553 | 24.3 |
|  | UK | 96 | 29.9 |  |  | Switzerland | 76 | 20.1 |
|  |  |  |  |  |  | UK | 96 | 20.6 |
| *N* | 2,897 | | |  |  | 2,897 | | |

*Note.* UK = United Kingdom.

In the first supplementary analysis, we conducted multi-level analyses using daily threat with country implemented as a third level. That is, instead of modelling country-level religiosity and societal marginalization as dummy-coded variables within 2-level models, they were included as level-3-predictors in 3-level models. We did not aim to include country as a third level in our main analyses due to sample size considerations (see Stegmueller, 2013) given the limited number of countries with sufficient participants. The results of the 3-level models are presented in Table C2 and are in line with the findings of our main analyses: We find a weaker relationship between RWA and threat perceptions in highly religious compared with little religious (b = −0.25 [−0.48, −0.02]) and in highly religious compared with moderately religious countries (−0.27 [−0.53, −0.04]) while not finding support for a moderation by moderately compared with little religious countries (0.03 [−0.16, 0.23]). Regarding the moderation by country-level marginalization, we find a weaker relationship between RWA and threat perceptions in highly marginalized compared with little marginalized (b = −0.23 [−0.54, 0.04]) and in highly marginalized compared with moderately marginalized countries (−0.28 [−0.62, −0.01]) while not finding support for a moderation in moderately marginalized compared with little marginalized countries (0.05 [−0.15, 0.25]).

**Table C2**

*Results of 3-Level Models (Supplementary Analysis 1) with countries with N > 50 (Study 3).*

|  | **Model 1** | | | |
| --- | --- | --- | --- | --- |
| **Predictor** | **b** | **95%-CI** | ***t*** | ***p*** |
| (Intercept) | **2.17** | [1.64, 2.71] | 7.36 | <.001 |
| Individual-Level RWA | **0.60** | [0.47, 0.73] | 8.96 | <.001 |
| Religiosity Country (high) (ref = low) | **1.20** | [0.45, 1.96] | 2.88 | .022 |
| Religiosity Country (mod) (ref = low) | 0.92 | [0.01, 1.83] | 1.82 | .112 |
| RWA*Religiosity Country (high) (ref = low) | **−0.25** | [−0.48, −0.02] | −2.10 | .036 |
| RWA*Religiosity Country (mod) (ref = low) | 0.03 | [−0.16, 0.23] | 0.30 | .765 |

|  | **Model 2** | | | |
| --- | --- | --- | --- | --- |
| **Predictor** | **b** | **95%-CI** | ***t*** | ***p*** |
| (Intercept) | **3.09** | [2.35, 3.83] | 7.58 | <.001 |
| Individual-Level RWA | **0.63** | [0.48, 0.77] | 8.38 | <.001 |
| Religiosity Country (high) (ref = mod) | 0.29 | [−0.63, 1.20] | 0.57 | .588 |
| Religiosity Country (low) (ref = mod) | −0.92 | [−1.83, −0.01] | −1.82 | .112 |
| RWA*Religiosity Country (high) (ref = mod) | **−0.27** | [−0.53, −0.04] | −2.26 | .024 |
| RWA*Religiosity Country (low) (ref = mod) | −0.03 | [−0.23, 0.16] | −0.30 | .765 |

|  | **Model 3** | | | |
| --- | --- | --- | --- | --- |
| **Predictor** | **b** | **95%-CI** | ***t*** | ***p*** |
| (Intercept) | **2.24** | [1.87, 1.57] | 10.90 | <.001 |
| Individual-Level RWA | **0.56** | [0.45, 0.67] | 10.08 | <.001 |
| Marginalization Country (high) (ref = low) | **1.57** | [0.86, 2.27] | 4.01 | .005 |
| Marginalization Country (mod) (ref = low) | **0.92** | [0.32, 1.53] | 2.76 | .029 |
| RWA*Marginalization Country (high) (ref = low) | −0.23 | [−0.54, 0.04] | −1.59 | .113 |
| RWA*Marginalization Country (mod) (ref = low) | 0.05 | [−0.15, 0.25] | 0.50 | .615 |

|  | **Model 4** | | | |
| --- | --- | --- | --- | --- |
| **Predictor** | **b** | **95%-CI** | ***t*** | ***p*** |
| (Intercept) | **3.17** | [2.69, 3.64] | 11.94 | <.001 |
| Individual-Level RWA | **0.62** | [0.45, 0.79] | 7.17 | <.001 |
| Marginalization Country (high) (ref = mod) | 0.64 | [−0.12, 1.41] | 1.51 | .173 |
| Marginalization Country (low) (ref = mod) | **−0.92** | [−1.53, −0.32] | −2.76 | .029 |
| RWA*Marginalization Country (high) (ref = mod) | −0.28 | [−0.62, −0.01] | −1.77 | .077 |
| RWA*Marginalization Country (low) (ref = mod) | −0.05 | [−0.25, 0.15] | −0.50 | .615 |

*Notes.* *N* = 2,897. RWA = right-wing authoritarianism. Unstandardized regression weights are reported. The outcome variable were threat perceptions towards people in one’s own country who belong to a different ethnic, religious, or national group than oneself. Bold numbers indicate significant coefficients. Individual-level RWA was mean-centered.

Our second supplementary analysis plan included a two-step approach: First, we calculated simple linear regression analyses for the prediction of threat perceptions by RWA individually for each country with N > 50 respondents. An overview of these countries and their values in religiosity and marginalization is given in Table C1.

Regarding religiosity and starting with trait threat, we found a positive RWA-Threat link of very large size for both moderately (β_trait, Italy_ = .46 [.34, .58], β_trait, Poland_ = .51 [.37, .65]) and little religious (β_trait, France_ = .54 [.40, .68], β_trait, Germany_ = .52 [.44, .60], β_trait, Switzerland_ = .53 [.26, .80], β_trait, UK_ = .41 [.16, .66]) countries. Among the four countries categorized as high in religiosity, three countries showed large to very large effect sizes (β_trait, Georgia_ = .32 [.08, .56], β_trait, South Africa_ = .54 [.29, .79], β_trait, Turkey_ = .40 [.15, .65]), and one country (Thailand) showed a non-significant relationship between RWA and threat perceptions (β_trait, Thailand_ = .05 [-.32, .42]). Using daily threat, we found small coefficients (≤.15) for the RWA-Threat link not reaching statistical significance in any of the four high-religiosity countries while countries moderate in religiosity (β_trait, Italy_ = .20 [.00, .40], β_trait, Poland_ = .29 [.04, .54]) and two out of four countries low in religiosity (β_trait, France_ = .38 [.18, .58], β_trait, Germany_ = .25 [.11, .39]) did show a significantly positive link of medium to large size.

Regarding marginalization and, again, starting with trait threat, we found a positive RWA-Threat link of very large size for little marginalized countries (β_trait, France_ = .54 [.40, .68], β_trait, Germany_ = .52 [.44, .60], β_trait, Italy_ = .46 [.34, .58], β_trait, Switzerland_ = .53 [.26, .80], β_trait, UK_ = .41 [.16, .66]) and a positive RWA-Threat link of large to very large size for moderately marginalized countries ((β_trait, Georgia_ = .32 [.08, .56], β_trait, Poland_ = .51 [.37, .65], β_trait, South Africa_ = .54 [.29, .79]). Among the two countries categorized as high in marginalization one country showed a positive RWA-Threat link of very large effect size (β_trait, Turkey_ = .40 [.15, .65]), while the other showed a non-significant relationship between RWA and threat perceptions (β_trait, Thailand_ = .05 [-.32, .42]). Using daily threat, we found small coefficients (≤.05) for the RWA-Threat link not reaching statistical significance in any of the two highly marginalized countries while one out of three moderately religious countries (β_trait, Poland_ = .29 [.04, .54]) and three out of five little marginalized countries (β_trait, France_ = .38 [.18, .58], β_trait, Germany_ = .25 [.11, .39], β_trait, Italy_ = .20 [.00, .40]) showed a significant RWA-Threat link of medium to large effect size.

As a second step, and merely for descriptive purposes, we then correlated the regression coefficients with the individual country values in religiosity and marginalization (that is, without categorizing them into low, moderate, high; see the index column of Table C1). We found negative correlations between the regression coefficients of the RWA-threat link and the country-individual indices of religiosity for both trait threat (*r_trait_* = -.32 [-.79, .39]) and daily threat (*r_daily_* = -.64 [-.91, -.02]) as well as between the regression coefficients of the RWA-threat link and the country-individual indices of marginalization, for both trait threat (r_trait_ = -.24 [-75., .46]) and daily threat (r_daily_ = -.50 [-.86, .19]). Given the very small number of countries in these supplementary analyses, we refrained from applying tests of significance and results, albeit being descriptively in line with our main analyses, should be interpreted with caution. Next, we provide a glance at the results of exploratory analyses regarding additional country-level indicators.

**Gross Domestic Product (GDP)**


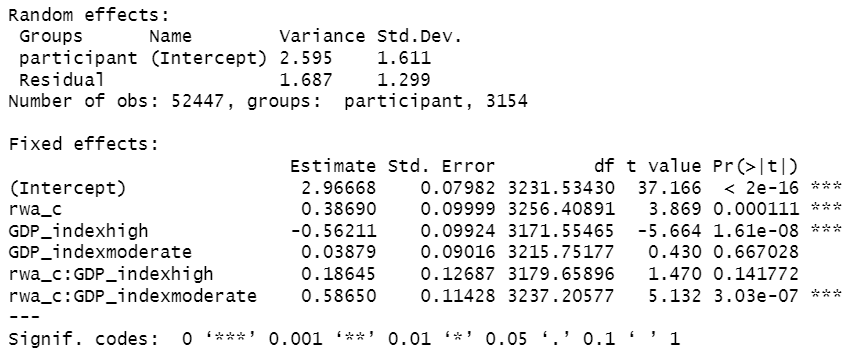
The specific GDP estimates were extracted from the World Development Indicators database, which is provided by the World Bank (2024b). We utilized the most recent GDP estimates that were available at the date of retrieval, which referred to the years 2021 or 2022, depending on the country. To obtain comparable values for all countries, we used GDPs based on purchasing power parities (PPPs). This approach was essential to account for the differences in price levels across economies and the varying purchasing powers of different currencies (World Bank, 2021). In short, GDPs (PPP) are GDPs converted into international dollars by using purchasing power rates. The purchasing power of an international dollar equals the purchasing power of a US dollar in the United States (World Bank, 2024a). Specifically, we used the GDP (PPP) estimates per capita based on the constant 2017 international dollar. The underlying PPP data are based on the International Comparison Program (ICP) managed by the World Bank and the Eurostat-OECD PPP Program (for further information, methodology and calculation of GDPs, see Eurostat & OECD, 2012; World Bank, 2021; World Bank, 2024a). We used the GDP data to divide the countries into three different groups with a low (<30.000 Int$), medium (30.000 to 50.000 Int$) and high (>50.000 Int$) GDP. As a result, 23 countries exhibited a low GDP, 10 countries a medium GDP and eight countries a high GDP. However, it should be noted that the GDP data were distributed rather continuously. Therefore, the categorial classification may be somewhat artificial.

| 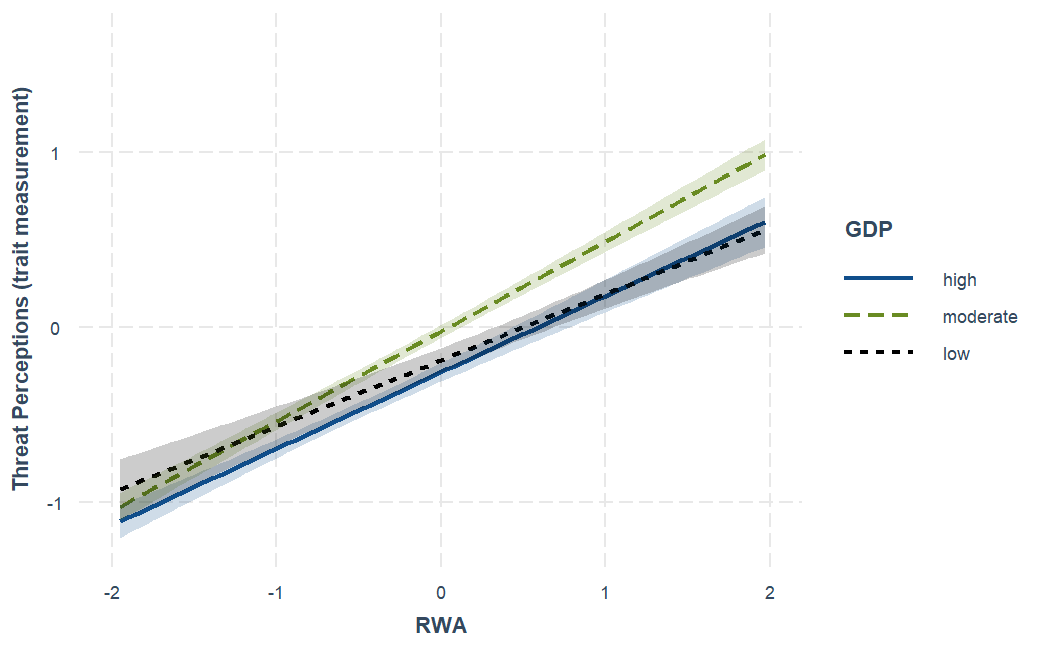  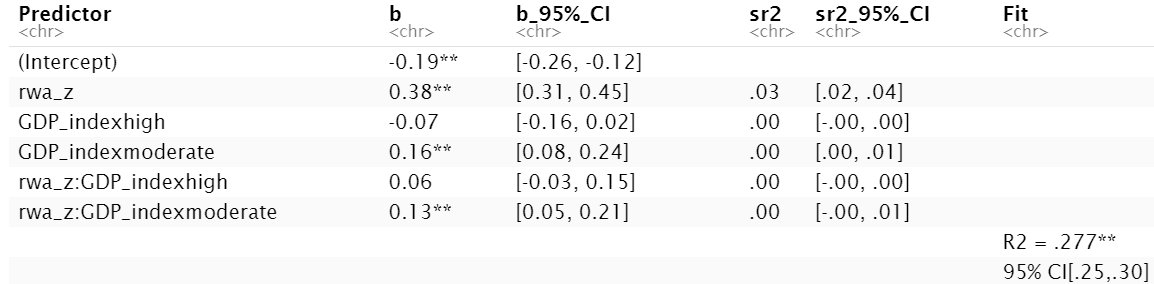 | 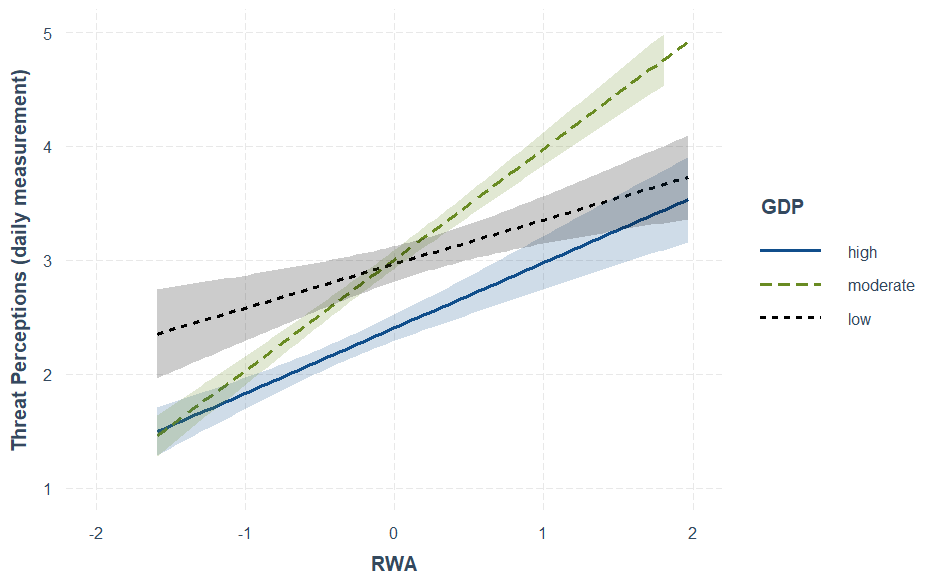 |
| --- | --- |

**Governmental Leaning: Populism**


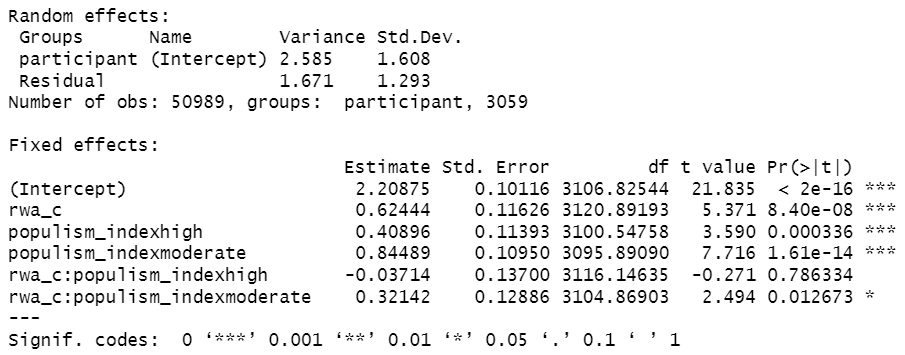

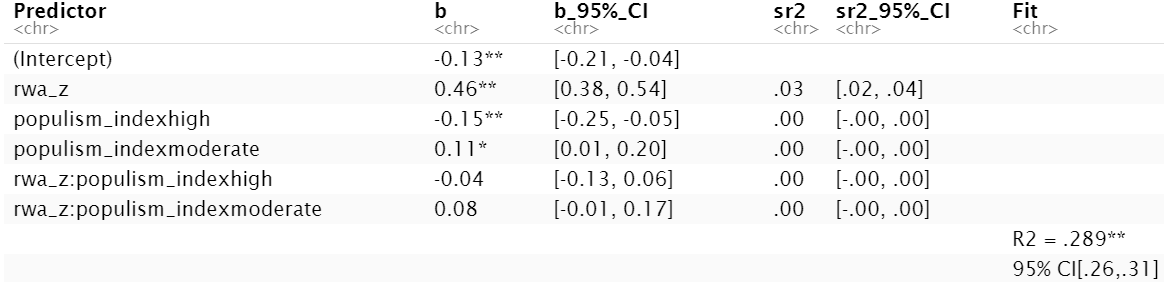
In order to obtain country scores for populism, we drew on the dataset of the 2019 Global Party Survey (GPS, Norris, 2020b, 2020c). This study comprises assessments from 1861 experts regarding 1043 political parties in 163 countries. In their assessments, the experts evaluated the extent to which political parties employed populist rhetoric. According to Norris (2020c, p. 702), “populist language typically challenges the legitimacy of established political institutions and emphasizes that the will of the people should prevail”. Along with a dimensional rating, this resulted in a categorical classification of the parties ranging from 1 (Strongly Pluralist) to 4 (Strongly Populist). However, we aimed to calculate a populism score for whole countries rather than individual parties. Therefore, we added up the share of seats of all parties classified as moderate (3) or strongly populist (4) for each country. Scores for the share of seats were provided by the GPS dataset based on the Election Guide of the International Foundation for Electoral Systems (IFES, see Norris, 2020a for more details). The party lists and scores mostly referred to legislative elections as close as possible to the survey’s commencement in November 2019 (Norris, 2020c). In total, data for both the populism rating and the share of seats were available for 25 of the countries examined by us. Additionally, we directly extracted the share of seats for Thailand (IFES, 2024) and Georgia (IFES, 2016, 2023) from the IFES Election Guide and calculated the share of seats manually (scores for populism were available in the GPS dataset). This allowed us to ultimately assign a populism score to 27 countries. Afterwards, these countries were divided into three different groups with low (<33.3%), medium (33,3% to 66.6%) and high (>66.6%) populism. Each group consisted of nine countries.

| 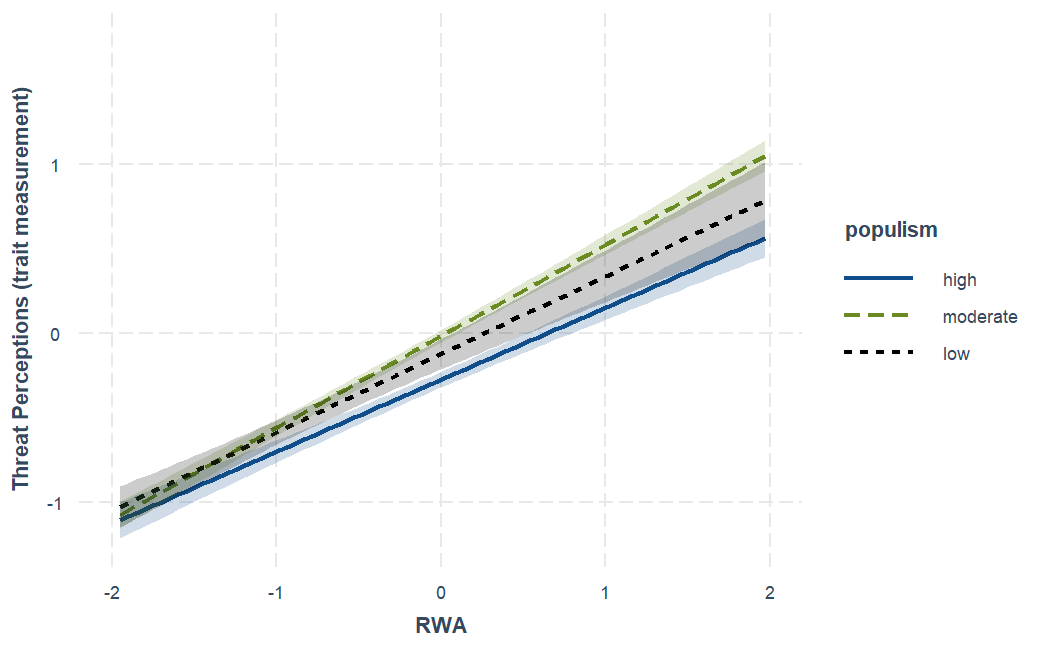 | 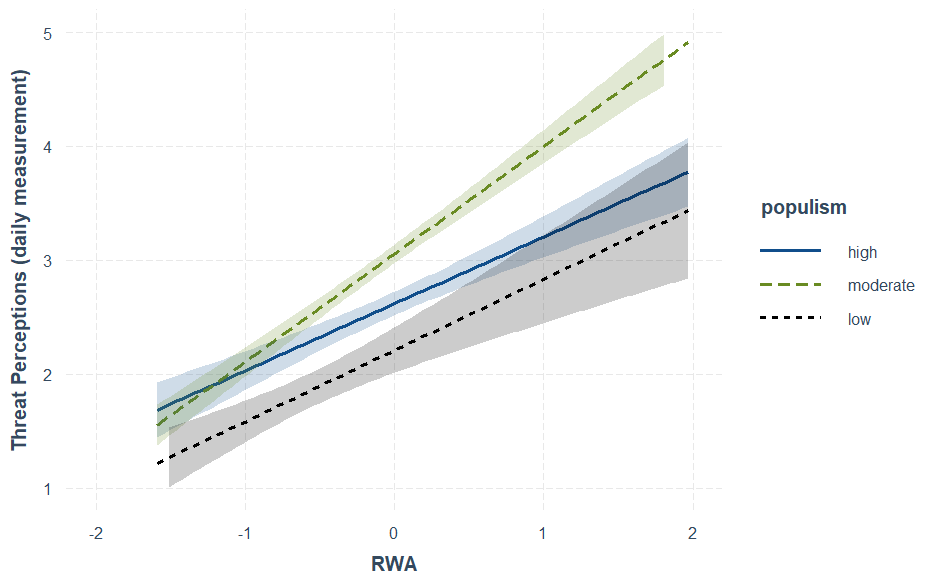 |
| --- | --- |

**Government Favoritism of Religion Index (GFI)**


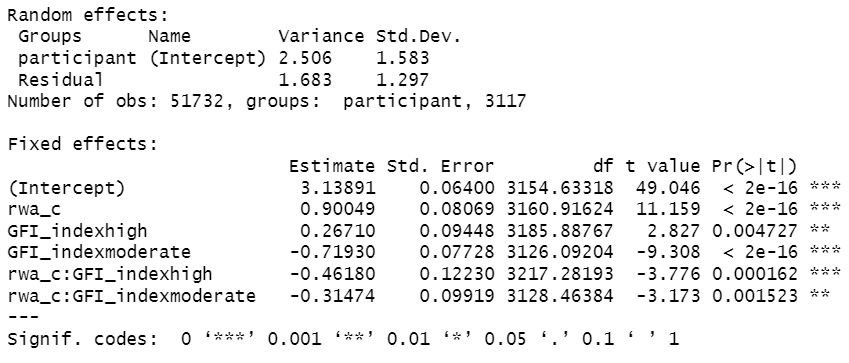

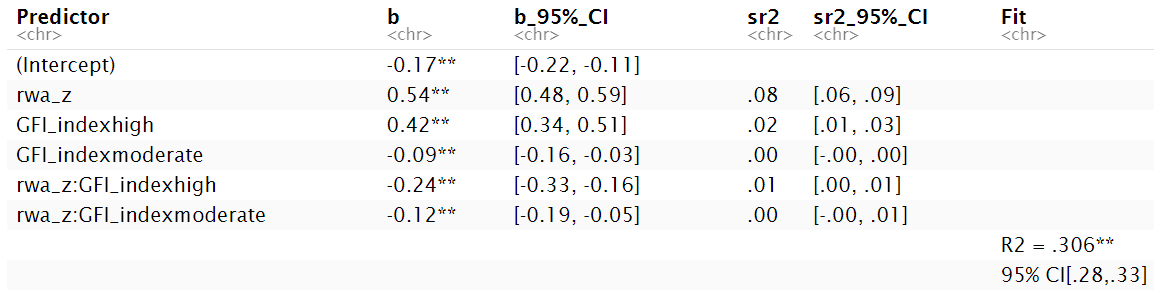
Furthermore, we made use of the Government Favoritism of Religion Index (GFI) developed by Grim and Finke (2006). Government favoritism can be defined as “actions of the state that provide one religion or a small group of religions with special privileges, support, or favorable sanctions” (Grim & Finke, 2006, p. 10). For example, this refers to disparate levels of government funding allocated to different religions. To generate this index, Grim and Finke (2006) coded data from the 2003 International Religious Freedom Report (U.S. State Department, 2003), which is based on each US embassy producing an annual report on religious freedom in their host country. These individual reports for each country were then reviewed by the US State Department (for more information on data collection and coding, see Grim & Finke, 2006). As declared by the authors, the State Department does not provide reports on regions under US control. Therefore, they were not able to assign a score to the United States in relation to this index. For all other examined countries, scores were available on a scale ranging from 0 to 10. Building on these scores, we divided the countries into three groups with low (<3), medium (3 to 6), and high (>6) GFI levels. Subsequently, 11 countries displayed a low GFI level, 10 countries a medium GFI level and 19 countries a high GFI level.

| 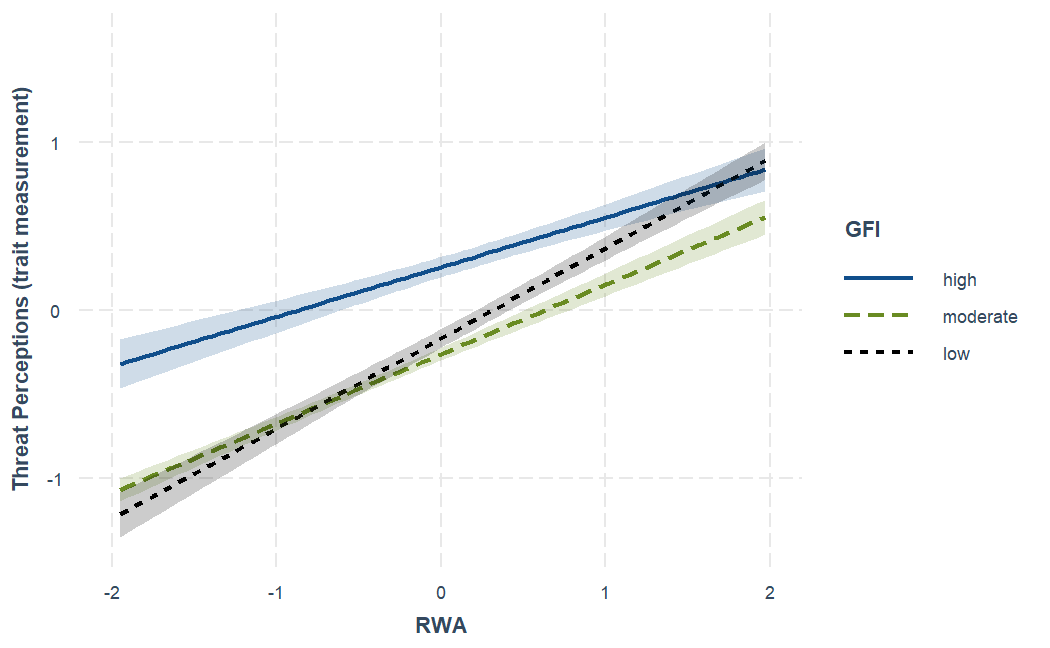 | 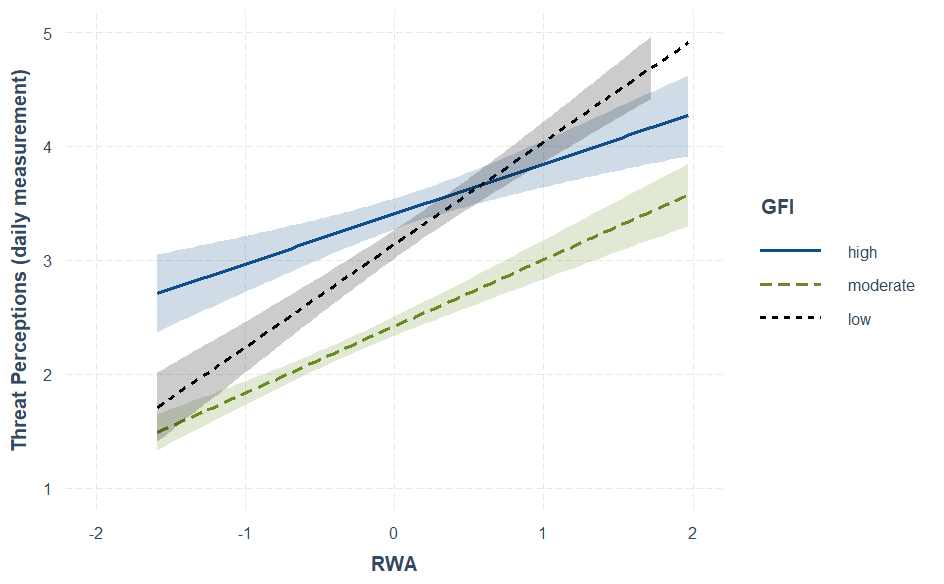 |
| --- | --- |

**Hofstede’s Individualism vs. Collectivism**


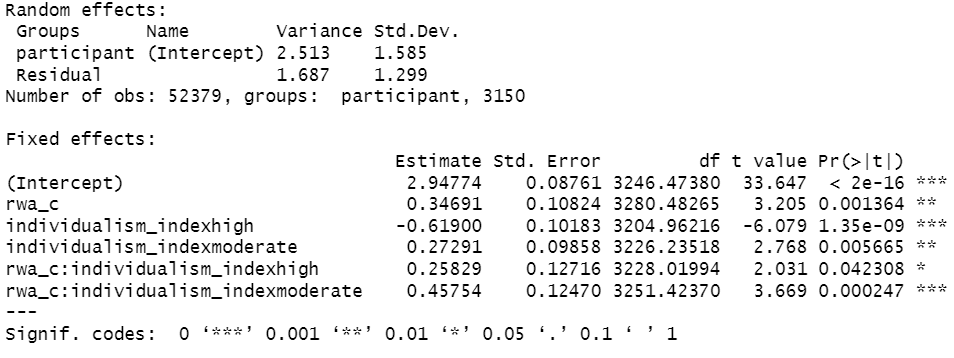

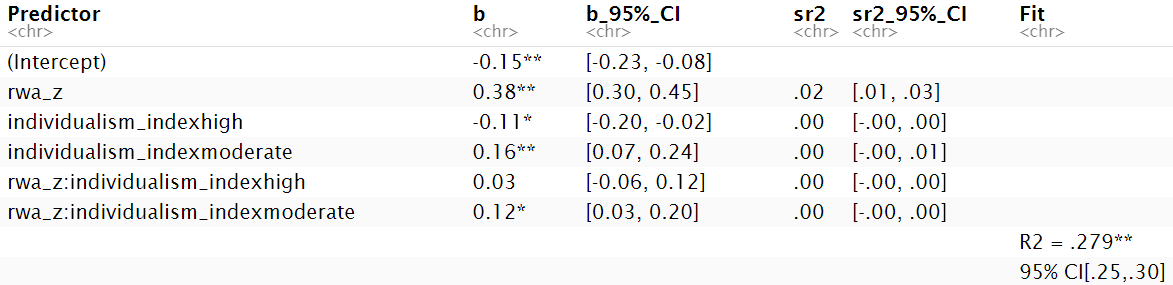
In order to derive a score for each country’s level of individualism, we employed the country comparison tool provided online by Hofstede Insights (2023). The online scores are updated periodically based on publications in scientific journals (e.g. Almutairi et al., 2021; Minkov & Kaasa, 2022). For countries not covered by publications, scores are derived from studies or commercial projects conducted by the Hofstede Insights research team. Nevertheless, no data was available for a few countries (i.e. Cameroon, Uganda and Zimbabwe). Scores range from 0 to 100, with higher values indicating a higher individualism. The construct of individualism, as opposed to collectivism, refers to “the degree to which people in a society are integrated into groups” (Hofstede, 2011, p. 11). While people in individualistic cultures tend to be loosely connected with each other, individuals in collectivistic cultures “are integrated into strong, cohesive in-groups”, such as “extended families” (Hofstede, 2011, p. 11). Again, we clustered the countries into three groups, with 15 countries having low (<40), 13 countries having medium (40 to 70), and 10 countries having high (>70) levels of individualism.

| 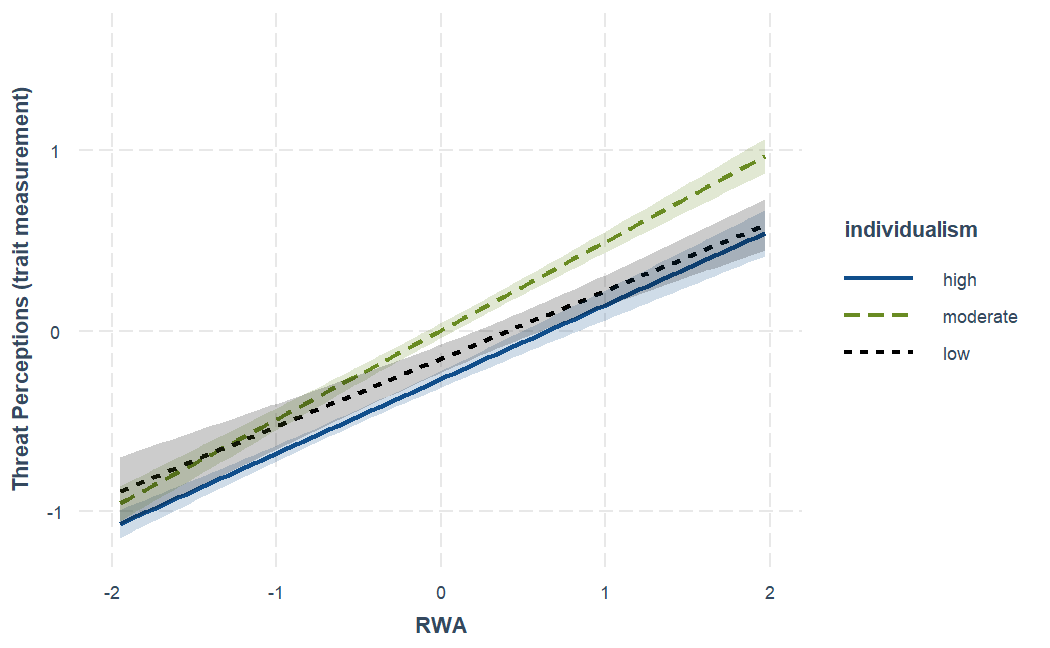 | 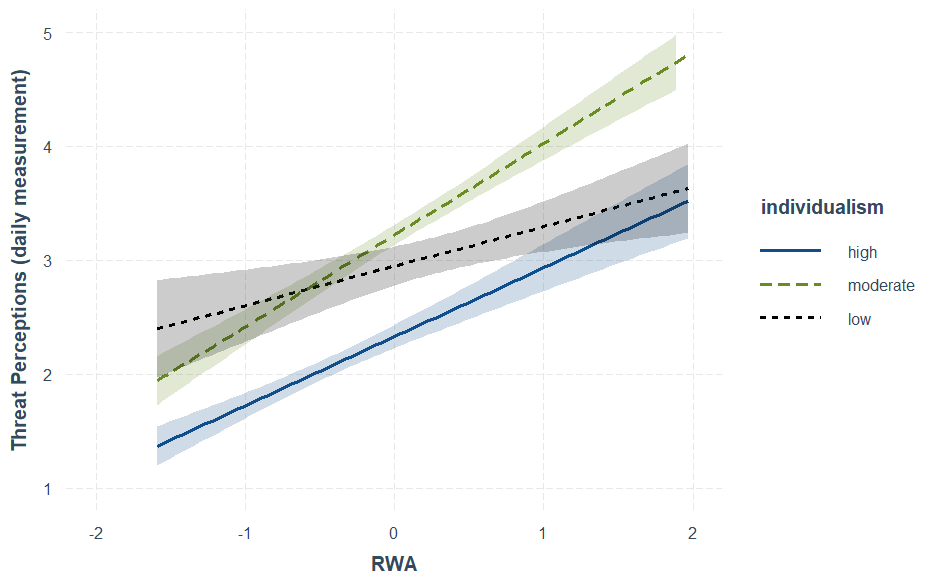 |
| --- | --- |

**Employment Rate**

Estimates for the employment rates were extracted from the database of the United Nations International Labour Organization (ILO) using the organization's official data exploration tool (ILO, 2023). More specifically, we used the annual employment-to-population ratio relating to the working age population (aged between 15 and 64 years). Regarding this population, estimates were available for all countries with the exception of China. The ILO data are based on various household surveys, predominantly labor force surveys. For more details on data collection and data sources for the specific countries, see ILO (2023) and ILO (n.d.). We again divided the countries into three groups. As a result, 17 countries displayed a low (<65%), 13 countries a medium (65% to 75%) and 10 countries a high (>75%) employment rate.

| 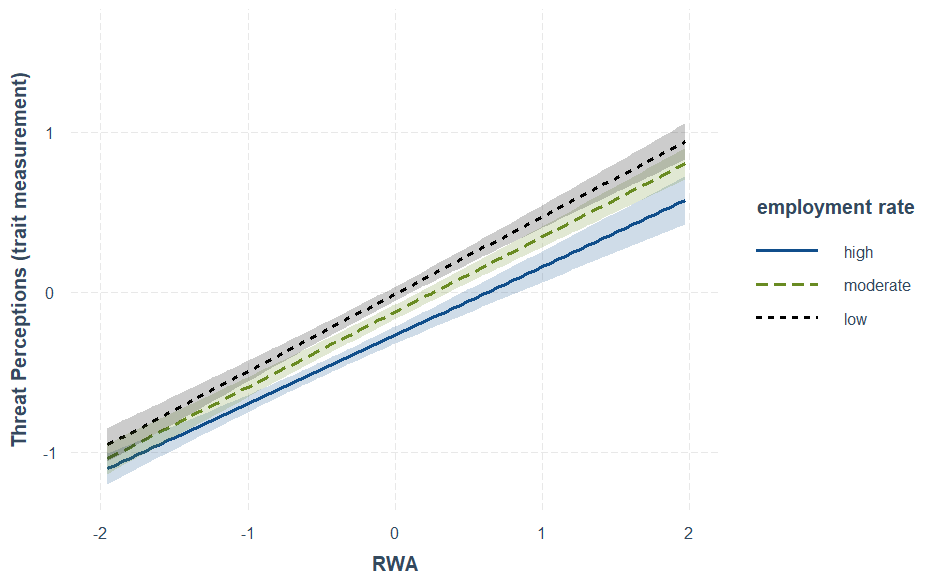 | 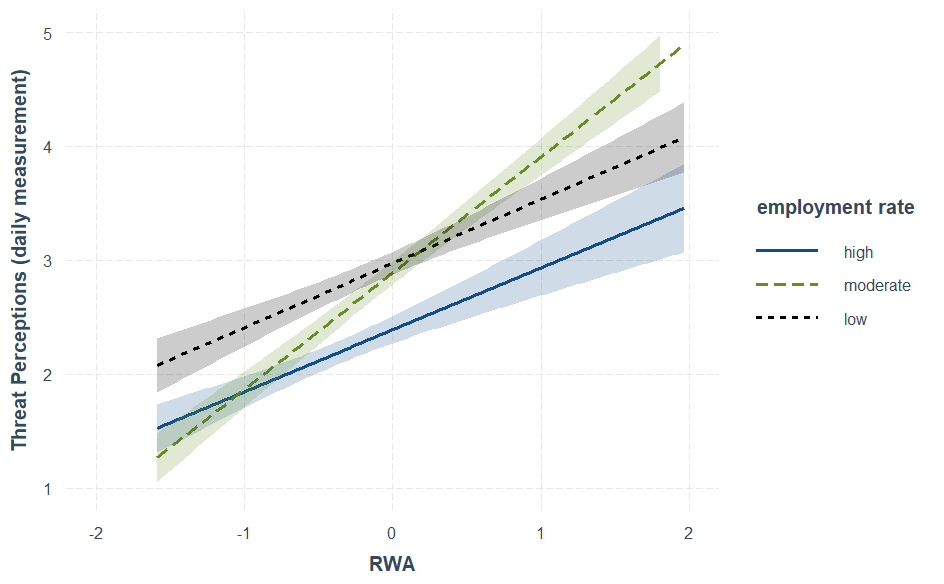 |
| --- | --- |


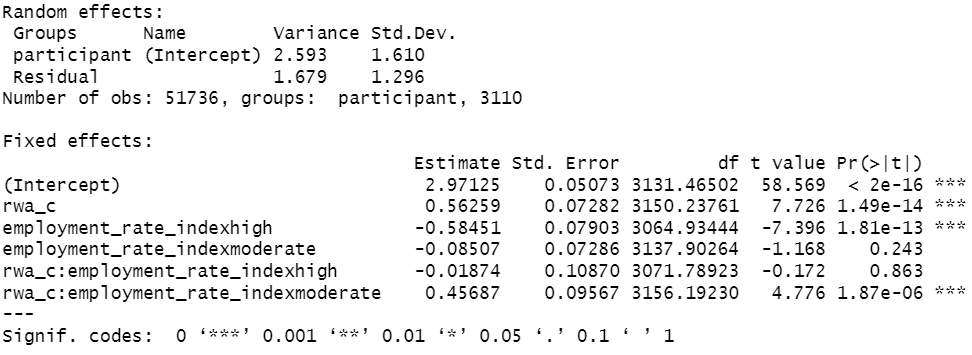

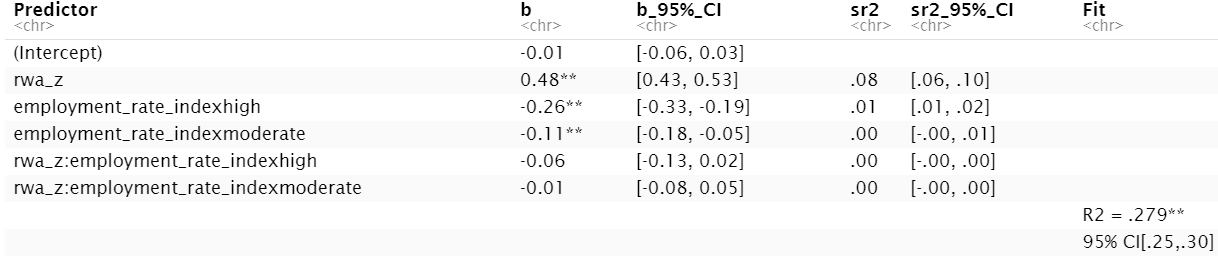


**International Migrant Stock**


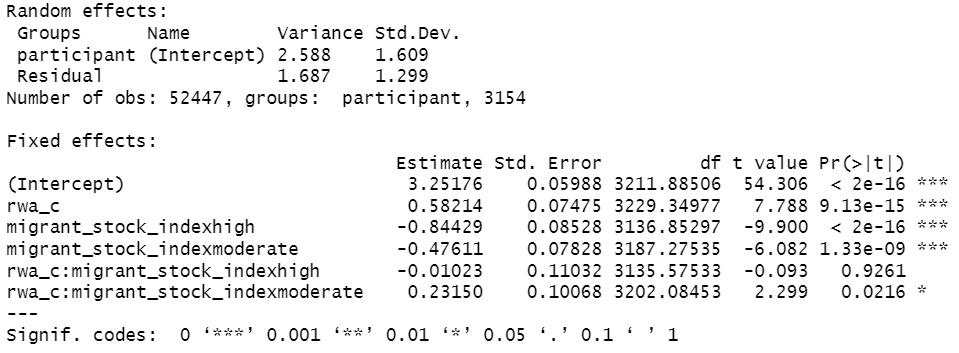
 Originally, we intended to use the Migrant Integration Policy Index (Solano & Huddleston, 2020) as a migration related index. However, data for this indicator was not available for many of the countries that are central to our analyses. Therefore, we employed the international migrant stock as an alternative indicator. Specifically, we referred to the proportion of international migrants as a percentage of the total population for both genders. The country estimates were derived from a publication based on the United Nations 2020 international migrant stock data set (United Nations Department of Economic and Social Affairs, Population Division, 2020a, 2020c). The underlying data mainly stem from population censuses. In addition, population registers and nationally representative surveys were used (for further information on methodology, see United Nations Department of Economic and Social Affairs, Population Division, 2020b). Based on the estimates, we split the countries into three groups with a low (<7%), medium (7% to 18%) and high (>18%) migrant stock. Accordingly, 22 countries exhibited a low, 10 countries a medium and nine countries a high migrant stock.

| 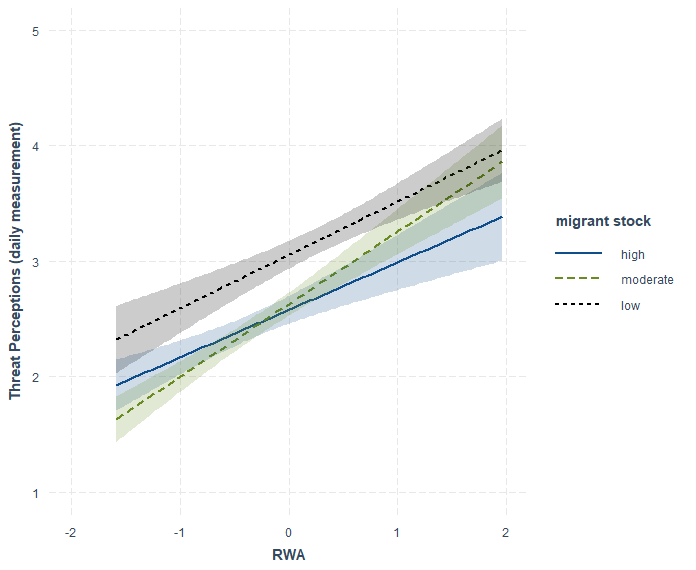 | 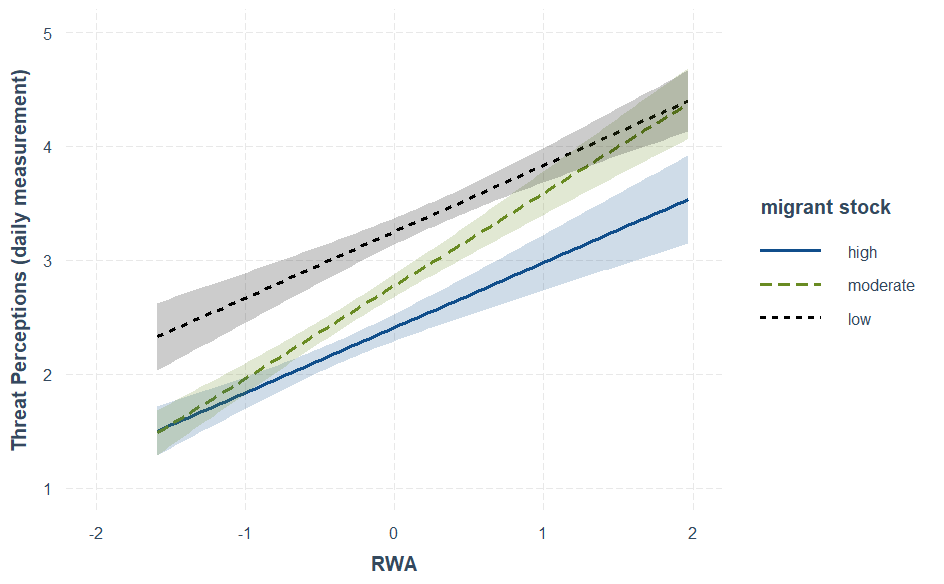 |
| --- | --- |


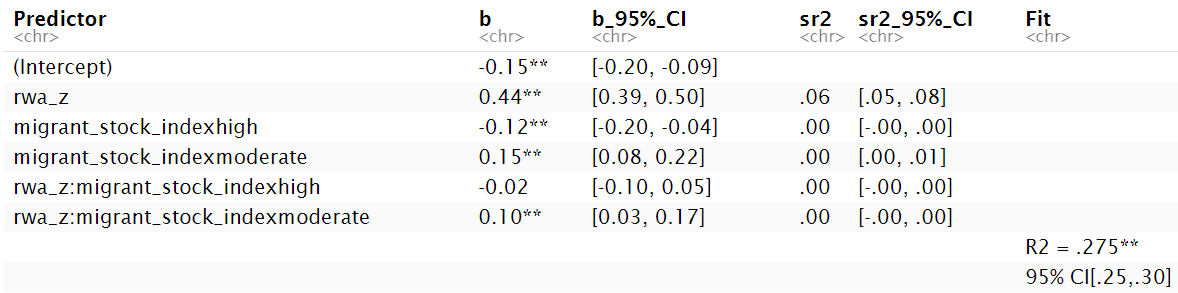


**References**

Almutairi, S., Heller, M., & Yen, D. (2021). Reclaiming the heterogeneity of the Arab states. *Cross Cultural & Strategic Management*, *28*(1), 158–176. <https://doi.org/10.1108/CCSM-09-2019-0170>

Eurostat, & OECD. (2012). *Eurostat-OECD methodological manual on purchasing power parities*. (Eurostat Methodologies and Working papers). <https://ec.europa.eu/eurostat/web/products-manuals-and-guidelines/-/ks-ra-12-023>

Grim, B. J., & Finke, R. (2006). International religion indexes: Government regulation, government favoritism, and social regulation of religion. *Interdisciplinary Journal of Research on Religion*, *2*, Article 1. <http://www.ncbi.nlm.nih.gov/pmc/articles/pmc4254791/>

Hofstede Insights. (2023, October 16). *Country comparison tool*. Retrieved June 20, 2024, from <https://www.hofstede-insights.com/country-comparison-tool>

Hofstede, G. (2011). Dimensionalizing cultures: The Hofstede model in context. *Online Readings in Psychology and Culture*, *2*(1), Article 8.
<https://doi.org/10.9707/2307-0919.1014>

International Foundation for Electoral Systems. (2016, October 13). IFES *ElectionGuide: Georgian Parliament 2016 general*. <https://www.electionguide.org/elections/id/2577/>

International Foundation for Electoral Systems. (2023, November 7). IFES *ElectionGuide: Georgian Parliament 2016 round 2*. <https://www.electionguide.org/elections/id/2989/>

International Foundation for Electoral Systems. (2024, May 10). *IFES ElectionGuide: Thai House of Representatives 2019 general*. <https://www.electionguide.org/elections/id/3139/>

International Labour Organization. (n.d.). *Data collection and production*. <https://ilostat.ilo.org/about/data-collection-and-production/>

International Labour Organization. (November 2023). *ILOSTAT data explorer: Employment-to-population ratio by sex and age (%) - annual*. Retrieved June 20, 2024, from <https://rshiny.ilo.org/dataexplorer6/?lang=en&id=EMP_DWAP_SEX_AGE_RT_A>

Minkov, M., & Kaasa, A. (2022). Do dimensions of culture exist objectively? A validation of the revised Minkov-Hofstede model of culture with World Values Survey items and scores for 102 countries. *Journal of International Management*, *28*(4), Article 100971. <https://doi.org/10.1016/j.intman.2022.100971>

Norris, P. (2020a). *Codebook: Global Party Survey, 2019*. Havard Dataverse. <https://doi.org/10.7910/DVN/WMGTNS/2WNIVR>

Norris, P. (2020b). *Global Party Survey, 2019: Global Party Survey by party SPSS V2_1_Apr_2020-1.tab* [Data set]. Havard Dataverse. <https://doi.org/10.7910/DVN/WMGTNS>

Norris, P. (2020c). Measuring populism worldwide. *Party Politics*, *26*(6), 697–717. <https://doi.org/10.1177/1354068820927686>

Solano, G., & Huddleston, T. (2020). *Migrant Integration Policy Index 2020.* Barcelona Center for International Affairs; Migration Policy Group. <https://www.mipex.eu/sites/default/files/downloads/pdf/files/a5/mipex-2020-book-a5.pdf>

Stegmueller, D. (2013). How many countries for multilevel modeling? A comparison of frequentist and Bayesian approaches. *American Journal of Political Science*, *57*(3), 748-761. <https://doi.org/10.1111/ajps.12001>

U.S. State Department. (2003). *2003 international religious freedom report*. <https://2009-2017.state.gov/j/drl/rls/irf/2003/index.htm>

United Nations Department of Economic and Social Affairs, Population Division. (2020a). *International migrant stock 2020* [Data set]. <https://www.un.org/development/desa/pd/sites/www.un.org.development.desa.pd/files/undesa_pd_2020_ims_stock_by_sex_and_destination.xlsx>

United Nations Department of Economic and Social Affairs, Population Division. (2020b). *Methodology report: International migrant stock 2020.* <https://www.un.org/development/desa/pd/sites/www.un.org.development.desa.pd/files/undesa_pd_2020_international_migrant_stock_documentation.pdf>

United Nations Department of Economic and Social Affairs, Population Division. (2020c). *International migration 2020 highlights.* <https://www.un.org/development/desa/pd/news/international-migration-2020>

World Bank. (2021). *Purchasing power parities for policy making: A visual guide to using data from the international comparison program.* <https://openknowledge.worldbank.org/handle/10986/35736>.

World Bank. (2024a, March 28). *DataBank | World development indicators: Metadata - preview* [Code: NY.GDP.PCAP.PP.KD]. Retrieved May 29, 2024, from [https://databank.worldbank.org/reports.aspx?source=2&type=metadata&series=NY.GDP.PCAP.PP.KD#](https://databank.worldbank.org/reports.aspx?source=2&type=metadata&series=NY.GDP.PCAP.PP.KD)

World Bank. (2024b, March 28). *World development indicators: GDP per capita, PPP (constant 2017 international $)* [Data set]. Retrieved May 29, 2024, from <https://data.worldbank.org/indicator/NY.GDP.PCAP.PP.KD>

1. *N* = 40 respondents have a value of -1 in the variable representing the total response time. This value is not defined. However, these respondents had no other conspicuous data, so that we kept them in the data set. [↑](#footnote-ref-1)
